# *Pwp1* regulates telomere length by stabilizing shelterin complex and maintaining histone H4K20 trimethylation

**DOI:** 10.1038/s41421-019-0116-8

**Published:** 2019-11-05

**Authors:** Yangyang Yu, Wenwen Jia, Yao Lyu, Dingwen Su, Mingliang Bai, Junwei Shen, Jing Qiao, Tong Han, Wenqiang Liu, Jiayu Chen, Wen Chen, Dan Ye, Xudong Guo, Songcheng Zhu, Jiajie Xi, Ruixin Zhu, Xiaoping Wan, Shaorong Gao, Jiyue Zhu, Jiuhong Kang

**Affiliations:** 1 Clinical and Translational Research Center of Shanghai First Maternity and Infant Hospital, Shanghai Key Laboratory of Signaling and Disease Research, Collaborative Innovation Center for Brain Science; School of Life Sciences and Technology, Tongji University, Shanghai, 200092 China; 2 Institute for Regenerative Medicine, Shanghai East Hospital, School of Life Sciences and Technology, Tongji University, Shanghai, 200123 China; 3 Department of Gynecology, Shanghai First Maternity and Infant Hospital, School of Medicine, Tongji University, Shanghai, 201204 P. R. China; 4 Department of Pharmaceutical Sciences, College of Pharmacy, Washington State University, Spokane, WA 99210 USA

**Keywords:** Telomeres, Embryonic stem cells

## Abstract

Telomere maintenance is critical for chromosome stability. Here we report that periodic tryptophan protein 1 (PWP1) is involved in regulating telomere length homeostasis. *Pwp1* appears to be essential for mouse development and embryonic stem cell (ESC) survival, as homozygous *Pwp1*-knockout mice and ESCs have never been obtained. Heterozygous *Pwp1*-knockout mice had shorter telomeres and decreased reproductive capacity. *Pwp1* depletion induced rapid telomere shortening accompanied by reduced shelterin complex and increased DNA damage in telomeric regions. Mechanistically, PWP1 bound and stabilized the shelterin complex via its WD40 domains and regulated the overall level of H4K20me3. The rescue of telomere length in *Pwp1*-deficient cells by PWP1 overexpression depended on SUV4-20H2 co-expression and increased H4K20me3. Therefore, our study revealed a novel protein involved in telomere homeostasis in both mouse and human cells. This knowledge will improve our understanding of how chromatin structure and histone modifications are involved in maintaining telomere integrity.

## Introduction

Telomeres are special structures located at chromosomal ends. They are composed of variable numbers of the simple DNA repeat TTAGGG, which is associated with large shelterin complex proteins^1^. This type of telomeric nucleoprotein structure is conserved in the majority of eukaryotic species. The variable numbers of telomeric DNA repeats result in differences in telomere length^2^. Telomeres are indispensable for chromosome stability in all species, including humans, mice, and ciliates^3^. Abnormal telomere shortening causes the development of telomere-related diseases, such as Dyskeratosis Congenital^4^. Therefore, elucidating the molecular mechanisms underlying the regulation of telomere length is particularly important. Currently, the known mechanisms of telomere length regulation^5,6^ are as follows: (1) telomerase-dependent telomere regulation, involving telomerase reverse transcriptase (TERT), RNA template (*Terc*), or other telomerase-associated factors^7^; (2) alternative lengthening of telomeres (ALT), involving proteins such as ZSCAN4^8,9^; (3) telomere protection by the shelterin complex, involving TRF1 (telomeric repeat-binding factor 1), TRF2 (telomeric repeat-binding factor 2), RAP1 (repressor and activator protein 1), POT1 (protection of telomeres 1), TPP1 (TIN2-interacting protein 1), and TIN2 (TRF1-interacting nuclear protein 2)^10^; (4) telomeric chromatin structure maintenance by the covalent modification of histones, such as H4K20me3 and H3K9me3^11^; and (5) telomere length regulation by non-coding RNA, such as TERRA^12,13^.

Previous studies have shown that telomeres have important functions in embryonic stem cells (ESCs)^14^. In *Tert*- or *Terc*-deficient ESCs, telomere length is decreased, genomic DNA is abnormally methylated, and H3K27me3 exhibits abnormal enrichment. These changes decrease the differentiation potential of ESCs and their ability to form teratomas and chimeras. Telomeres rapidly shorten upon ESC differentiation and gradually lengthen during the reprogramming of somatic cells into induced pluripotent stem cells^15^. *Zscan4*, identified as a specific marker for two-cell embryos and ESCs, is involved in telomere maintenance and long-term genomic stability in ESCs^16^. Overexpression of ZSCAN4 in ESCs rapidly increases telomere length through telomeric sister chromatid exchange (T-SCE). Decreasing *Zscan4* expression through RNA interference decreases telomere length and proliferation of ESCs after passaging for 7–8 generations^16–18^.

The shelterin complex plays an important role in remodeling telomeric structures^19^. TRF1 and TRF2, two telomeric repeat-binding proteins in the shelterin complex, are abundant in telomeric regions and interact with telomeric repeats and other proteins, facilitating the formation of telomeric loops and the synthesis of short telomere-like small fragments^20–23^. POT1 proteins bind primarily to the single-stranded region of telomeric DNA^24^. In mice, POT1a inhibits ATR kinase-mediated telomere signals, and POT1b regulates the protruding end of the single-stranded telomeric DNA. Heterozygous *Pot1b*-knockout mice develop normally, but homozygous *Pot1a-*knockout mice is embryonic lethal^25,26^. Therefore, the shelterin complex is a critical regulator for maintaining telomere integrity.

The histone marks H3K9me3 and H4K20me3 are highly enriched in subtelomeric and telomeric regions^11^. Mouse embryonic fibroblasts from telomerase-deficient mice have shortened telomeres and reduced H3K9me3 and H4K20me3 in subtelomeric and telomeric regions^27^. However, it has also been shown that after *Suv4-20h2* knockout, the H4K20me3 mark is significantly decreased in telomeric and subtelomeric regions, but the telomere length increases^28^. Abnormal DNA methylation and reduced H3K9me3 and H4K20me3 have been found in telomeric and subtelomeric regions in cancer cells. These changes might help to activate the telomere elongation mechanism and maintain the proliferative capacity of cells that have lost telomerase activity. These data have exposed the complexity of telomere length regulation by histone modifications in subtelomeric and telomeric regions^28,29^. Moreover, the molecular mechanism underlying the regulation of telomere length in ESCs also awaits further investigation. In particular, few studies have been performed to understand how histone modifications collaborate with the shelterin complex in telomere length regulation.

Proteins with the WD40 domain have a wide variety of biological functions. They are involved in the stabilization of protein complexes, RNA processing, DNA replication, transcriptional regulation, the maintenance of genome stability, histone modifications, cell cycle regulation, and tumor development^30,31^. For example, WD repeat domain 5 (WDR5), a core component of the TrxG complex, acts as an effector molecule of H3K4 methylation to regulate the self-renewal of ESCs^32^. Periodic tryptophan protein 1 (PWP1) is a typical WD40 repeat protein^33^. Our previous studies indicated that this protein affected the multipotent differentiation capacity of ESCs by influencing the level of H4K20me3^34^. Here, we report that *Pwp1* is present in mouse testicular tissues, where telomere lengthening mainly occurs. Mice with heterozygous *Pwp1*-knockout exhibited significant telomere shortening accompanied by a reduced reproductive capacity. RNA interference-mediated *Pwp1* silencing resulted in a decrease in the accumulation of shelterin and H4K20me3 in telomeric regions and induced rapid telomere shortening.

## Results

### *Pwp1* depletion shortened telomere length

**Fig. 1:**
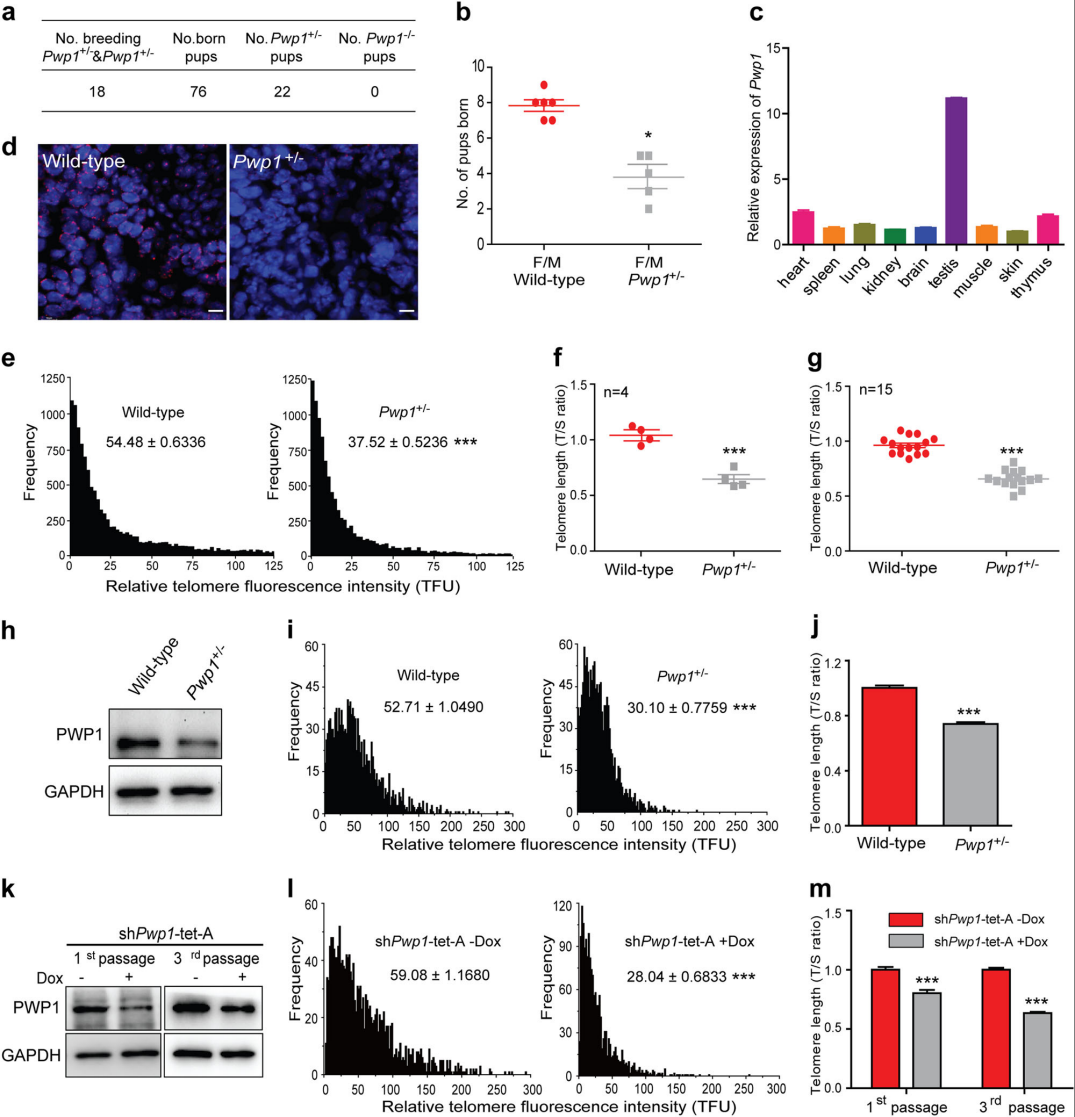
*Pwp1* is required to maintain telomere length. See also Supplementary Fig. S1. **a** Numbers of pups born by breeding *Pwp1*^+/−^ male and female mice. **b** Numbers of pups obtained by breeding wild-type and *Pwp1*^+/−^ mice. **c** *Pwp1* mRNA levels in mouse tissues. **d** Telomere Q-FISH images of wild-type and *Pwp1*^+/−^ mouse testes. Mouse testes were stained for telomeres (TTAGGG; red) and nuclei (DAPI; blue). The scale bar represents 10 μm. **e** Q-FISH analysis of telomere length in mouse testicular tissues. Telomere fluorescence intensity (TFU) determined by telomere Q-FISH. Shown are diagrams of the relative telomere length distribution in wild-type and *Pwp1*^+/−^ mouse testes. **f** qPCR analysis of telomere length in mouse testicular tissues. Assays were performed using four pairs of mice. **g** qPCR analysis of telomere length in mouse tails. Assays were performed using 15 pairs of mice. **h** PWP1 protein levels in wild-type and *Pwp1*^+/−^ mouse ESCs. **i** Q-FISH analysis of telomere length in ESCs. The data are shown as TFU determined by telomere Q-FISH in wild-type and *Pwp1*^+/−^ ESCs. **j** qPCR analysis of telomere length in ESCs. The data are shown as the T/S ratio determined by qPCR in wild-type and *Pwp1*^+/−^ ESCs. **k** Knockdown of PWP1 protein expression in ESCs. ESCs expressing sh*Pwp1* (sh*Pwp1*-tet-A) were passaged in the absence or presence of doxycycline (Dox). **l** Effect of *Pwp1* knockdown on telomere length in ESCs as measured by Q-FISH. Telomere lengths in sh*Pwp1*-tet-A ESCs at passage 1 were determined by telomere Q-FISH. **m** Effect of *Pwp1* knockdown on telomere length in ESCs as measured by qPCR. The data are shown as the T/S ratio determined by qPCR in sh*Pwp1*-tet-A ESCs at passages 1 and 3. The data are presented as the mean ± SEM of three independent experiments. *P < 0.05, ***P < 0.001

Our previous studies showed that *Pwp1* regulated the differentiation of mouse ESCs by inhibiting the LIF/Stat3 signaling pathway^34^. In addition, we detected high levels of *Pwp1* mRNA expression in the 2-cell stage of mouse embryonic development (Supplementary Fig. S1a, b). To better understand the function of *Pwp1* in mouse embryonic development, we construct *Pwp1*-knockout mice using the CRISPR/Cas9 method. Of the 180 embryos that received the plasmid containing a *Pwp1* gRNA, only six mice were born. In comparison, of the 120 embryos that received control plasmids, 48 mice were successfully born. As determined by PCR-DNA sequencing, among these six mice, two mice had the *Pwp1*^+/−^ genotype, and no mice were *Pwp1*^−/−^ (Supplementary Fig. S1c). One of these *Pwp1*^+/−^ mice contained a 5-bp deletion, resulting in a frameshift in the *Pwp1* coding region (Supplementary Fig. S1d). When *Pwp1*^+/−^ mice were used for crossbreeding, there were only offspring with wild-type and *Pwp1*^+/−^ phenotypes, and no mice with the *Pwp1*^−/−^ phenotype were obtained (Fig. 1a). The offspring birth rate of *Pwp1*^+/−^ mouse breeding was also significantly lower than that of wild-type mice (Fig. 1b), indicating that the *Pwp1* mutation affected embryonic development and embryo survival. Thus, we used mouse embryos to establish ES cell lines. Only 6 *Pwp1*^+/−^ cell lines were obtained from a total of 20 *Pwp1*^+/−^ embryos, and no *Pwp1*^−/−^ cell line was found (Supplementary Fig. S1e). In contrast, four cell lines were obtained from five wild-type embryos. In addition, we attempted to generate *Pwp1*-knockout ESCs using the CRISPR/Cas9 method. From the 56 ES clones screened, 10 *Pwp1*^+/−^ cell lines were obtained, but no *Pwp1*^−/−^ cell lines were found (Supplementary Fig. S1f). Our previous studies showed that *Pwp1* was required for the exit of ESCs from the pluripotent state into all lineages^34^. Together, our data suggested that *Pwp1* was essential for mouse embryonic development and ESC survival.

To determine the role of *Pwp1* in mouse development, we examined its mRNA levels in several mouse tissues by RT-qPCR. As shown in Fig. 1c, *Pwp1* was expressed at the highest level in the testes (Fig. 1c), suggesting that it played a role in spermatogenesis and reproduction. Interestingly, ~15% of *Pwp1*^+/−^ mice exhibited a congenital absence of facial hair (Supplementary Fig. S1g) similar to mouse models of Dyskeratosis Congenita with mutations in genes of telomerase or the shelterin component *Tin2*^35,36^. PWP1, as a chromatin-binding protein but not telomere specific, was widely expressed in the nucleus and also indeed localized at the telomere region (Supplementary Fig. S1h, i). Together with the telomere-specific ChIP-dot blot assays, we indeed detected an association between PWP1 and telomeres which determined the presence of PWP1 at telomeres (Supplementary Fig. S1j, k). Furthermore, PWP1 bound with the subtelomeric regions of chromosomes in mouse ESCs (Supplementary Fig. S1l). These results suggested that PWP1 could have an ability to regulate telomere homeostasis. To determine whether *Pwp1* is involved in telomere homeostasis, we measured the telomere length in testicular tissues and in the tails of wild-type and *Pwp1*^+/−^ mice using Q-FISH (Fig. 1d, e) and quantitative PCR (qPCR) methods (Fig. 1f, g). The results showed that the telomeres in the testes and tails of *Pwp1*^+/−^ mice were significantly shorter than those in wild-type mice, suggesting that *Pwp1* regulated telomere homeostasis.

In addition, telomeres were also shorter in *Pwp1*^+/−^ ESCs than in wild-type ESCs (Fig. 1h–j and Supplementary Fig. S1m). We therefore constructed ESC lines with an inducible *Pwp1* small hairpin RNA. Upon addition of doxycycline (Dox), both *Pwp1* mRNA and protein levels were down-regulated in ESCs of the first (2 days) and third (6 days) passages (Fig. 1k and Supplementary Fig. S1n). Accordingly, telomere length was markedly reduced within 48 h after *Pwp1* knockdown (Fig. 1l, m). Therefore, decreased *Pwp1* expression resulted in telomere shortening both in the mouse testis in vivo and in ESCs in vitro.

### Telomere shortening induced by *Pwp1* deficiency was independent of the expression of telomerase and *Zscan4*, and accompanied by reduced telomerase-telomere association

Telomere length homeostasis can be mediated by activating telomerase or other telomere regulatory factors^6^. Dox-induced *Pwp1* knockdown in ESCs did not cause changes in the expression of *Tert, Terc* or telomerase activity (Supplementary Fig. S2a–c). No change of TERT-*Terc* association was observed following *Pwp1* knockdown (Supplementary Fig. S2d). However, *Pwp1* depletion had a weak effect on telomerase-telomere association (Supplementary Fig. S1e, f). To further verify the role of telomerase on telomere shortening in *Pwp1* depletion ESC, we knocked down *Pwp1* in *Terc*^−/−^ ESCs. In the *Terc*^−/−^&sh*Pwp1*-tet-A ESCs, the reduction of *Pwp1* did cause telomere length shortening (Supplementary Fig. S1g, h). These indicted that the telomere shortening induced by *Pwp1* deficiency was not owing to telomerase. In addition, we also examined the changes in *Zscan4*, a key molecule in ALT^16,17,37,38^, in *Pwp1*-depleted cells. *Pwp1* knockdown induced the downregulation of ZSCAN4 protein (Supplementary Fig. S2i). However, telomere shortening was not detectable after *Zscan4* knockdown for 48 h (Supplementary Fig. S2j, k), and ZSCAN4 overexpression did not restore the shortened telomeres in *Pwp1*^+/−^ ESCs (Supplementary Fig. S2l, m). Thus, regulation of neither telomerase nor *Zscan4* was a major factor in the telomere shortening induced by *Pwp1* deficiency.

### Telomeric enrichment of shelterin complex was reduced in *Pwp1*-depleted ESCs

**Fig. 2:**
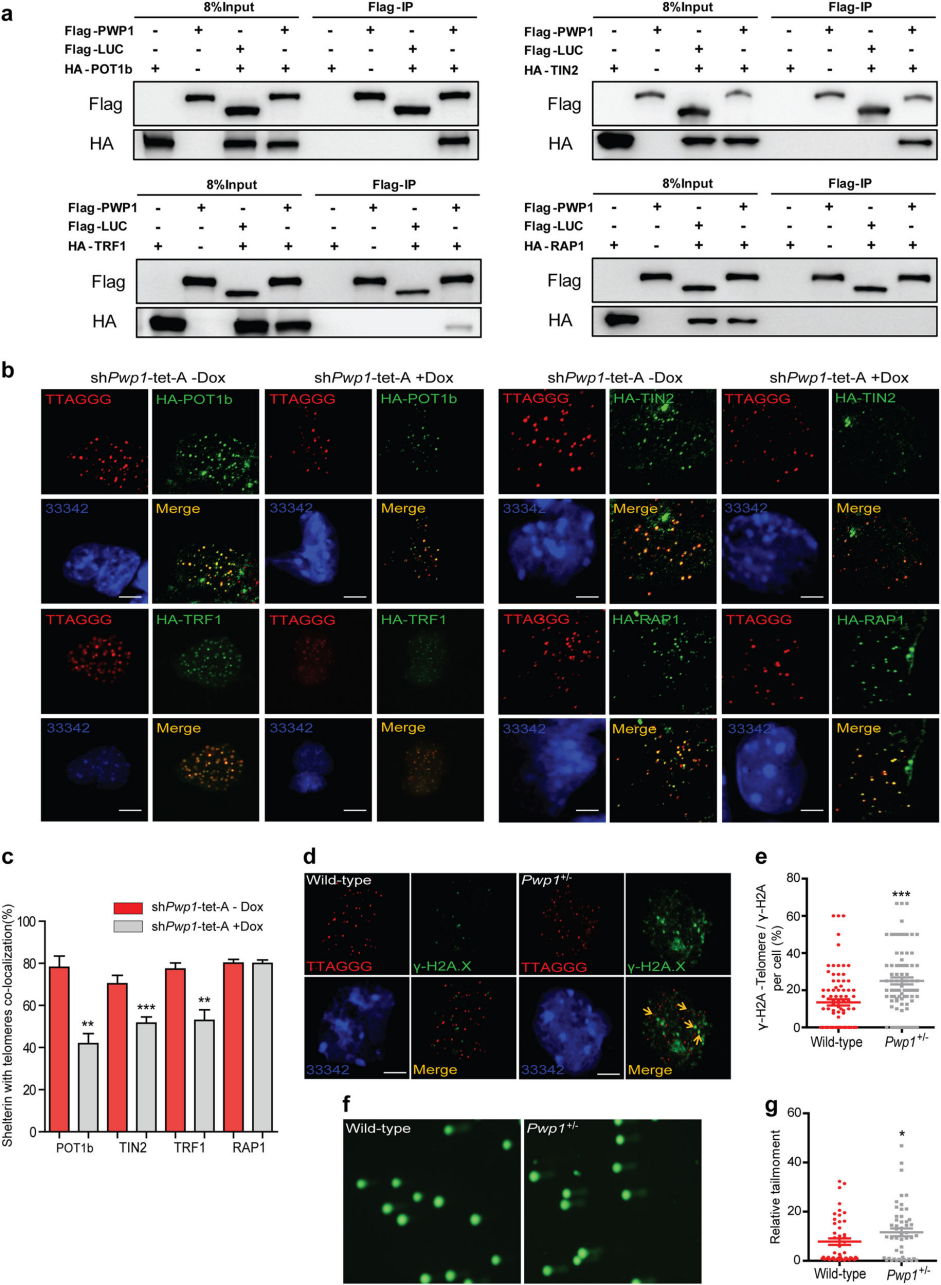
*Pwp1* depletion reduced shelterin complex enrichment at telomeres. See also Supplementary Fig. S2–S4. **a** Binding between PWP1 and the shelterin proteins. Plasmids expressing Flag-PWP1, Flag-Luciferase, and HA-shelterin were transfected into 293FT cells for 48 h. Cell extracts were immunoprecipitated using anti-Flag antibody, followed by Western blotting with an anti-HA antibody. **b**, **c** Effects of *Pwp1* knockdown on telomere recruitment of shelterin proteins in ESCs. ESCs containing sh*Pwp1*-tet-A were treated with Dox or without for 48 h and stained for telomeres (TTAGGG; red), shelterin (POT1b, TIN2, TRF1, and RAP1; HA antibody; green), and nuclei (Hoechst 33342; blue). **b** Representative immunofluorescence (IF)-FISH images. **c** Quantifications of co-localizing foci between shelterin proteins (POT1b, TIN2, TRF1, and RAP1) and telomeres. **d**, **e** Localization of γ-H2A.X in telomric region. Wild-type and *Pwp1*^+/−^ ESCs were subjected to IF-FISH analysis using a telomere probe (red) and an antibody against γ-H2A.X (green). **d** Representative images of γ-H2A.X-Telomere. **e** Quantification of co-localizing foci of γ-H2A.X and telomeres. The graph shows the percentage of γ-H2A.X-Telomere co-localizing foci among the total γ-H2A.X foci per cell. **f**, **g** DNA damage in wild-type and *Pwp1*^+/−^ ESCs. Representative comet assay images (**f**). Quantification of DNA damage as measured by the comet assay (**g**). The scale bar represents 10 μm. The data are presented as the mean ± SEM of three independent experiments. *P < 0.05, **P < 0.01, ***P < 0.001

The shelterin complex consists of a group of proteins at the telomere ends and functions to protect telomeres from DNA damage^19,39^. To investigate its role in *Pwp1* deficiency-induced telomere shortening, we performed the reciprocal co-immunoprecipitation (co-IP) to examine the association between PWP1 and six shelterin proteins (POT1 has two subunit structures, POT1a and POT1b, in mice^25^). PWP1 bound strongly to hemagglutinin (HA)-tagged POT1a, POT1b, TIN2, and TPP1, weakly to TRF1, but did not to TRF2 or RAP1 (Fig. 2a and Supplementary Fig. S3a, b). To further verify the shelterin binding with PWP1, we constructed two cell lines: (1) *Pot1*-knockdown with PWP1 and TIN2 over-expression ESCs; (2) *Tin2*-knockdown with PWP1 and POT1b over-expression ESCs. In our results, there were no significant binding changes in the both cell lines. These results showed that the binding of PWP1 and POT1b or PWP1 and TIN2 was not affected by *Tin2* depletion or *Pot1* depletion (Supplementary Fig. S3c, d). Next, plasmids expressing HA-tagged shelterin proteins (POT1b, TIN2, TRF1, and RAP1) were introduced into sh*Pwp1*-tet-A ESCs. After induction of sh*Pwp1* by Dox for 48 h, the enrichment of shelterin proteins (POT1b, TIN2, and TRF1) at telomeres was markedly decreased, whereas the telomere localization of RAP1, which did not bind to PWP1, did not change significantly (Fig. 2b, c). To determine the effect of *Pwp1* knockdown on endogenous shelterin proteins, an 3xHA tag was knocked into the carboxyl terminus of POT1b protein at the endogenous POT1b locus in the sh*Pwp1*-tet-A ESC line, allowing us to track the endogenous POT1b protein. In this cell line, the enrichment of endogenous POT1b protein at telomeres decreased in response to Dox treatment (Supplementary Fig. S4a, b). The reduction of shelterin enrichment at telomeres was corroborated by a telomere-specific ChIP-dot blot assay showing reduced association between POT1 (Supplementary Fig. S4c, d). In addition, we examined the expression of shelterin complex proteins in sh*Pwp1*-tet-A ESCs. Both mRNA and protein levels of shelterin complex proteins were reduced after *Pwp1* depletion (Supplementary Fig. S4e, f). Next, the DNA damage signals at the telomeric regions, as measured by γ-H2A.X staining and telomere-specific ChIP-dot blot assays, the DNA damage signals at the subtelomeric regions, as measured by ChIP-qPCR, and the overall levels of DNA damage, as determined by comet electrophoresis, significantly increased in *Pwp1*^+/−^ ESCs compared with those in wild-type cells (Fig. 2d–g and Supplementary Fig. S4g-i). Furthermore, we measured the telomere aberrations in our experiments. Among the four types of telomere aberrations, there were only slightly more terminal deletions after *Pwp1* reduction (Supplementary Fig. S4j, k). Therefore, our data indicated that decreases in *Pwp1* expression resulted in a reduction of shelterin complex assembly at telomeric regions, attenuating its telomere protective function.

**Fig. 3:**
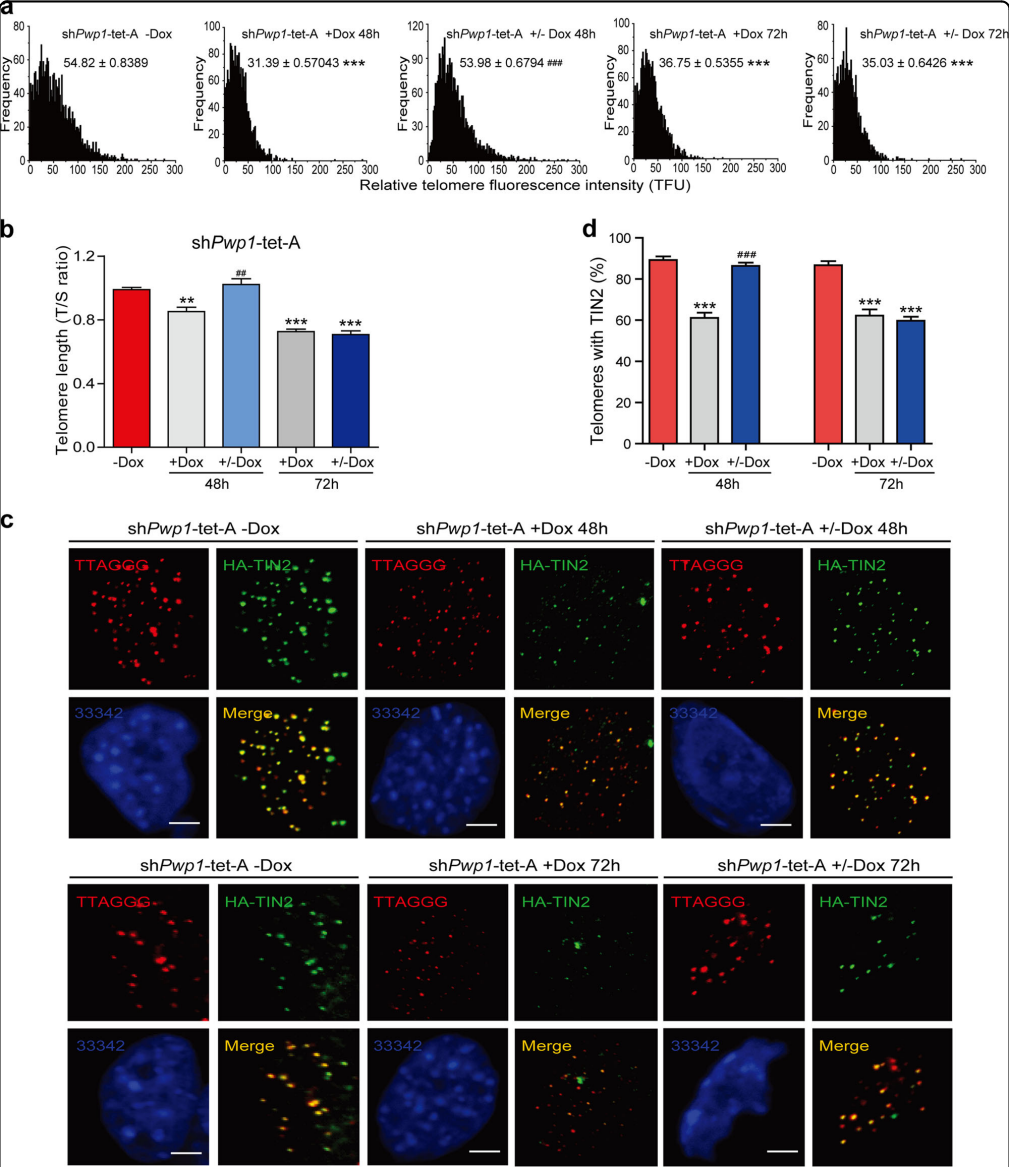
Only *Pwp1* could not rescue telomere length in prolonged-*Pwp1*- depletion ESCs. See also Supplementary Fig. S5. **a**, **b** Telomere length regulation by *Pwp1* knockdown. sh*Pwp1*-tet-A ESCs were treated with 1 µg/ml Dox for 48 or 72 h, and telomere length was determined by telomere Q-FISH. -Dox, no Dox treatment; +Dox, treated with Dox for 48 h or 72 h; +/− Dox, treated with Dox for 48 h or 72 h and then without Dox for 48 h or 72 h. **a** The data are shown as TFU determined by telomere Q-FISH. **b** The data are shown as the T/S ratio determined by telomere qPCR. **c**, **d** Shelterin formation in *Pwp1*-knockdown cells. sh*Pwp1*-tet-A ESCs were treated with Dox for 48 h or 72 h. Cells were stained for telomeres (TTAGGG; red), shelterin (TIN2; green), and nuclei (Hoechst 33342; blue). **c** Representative images. −Dox, without Dox treatment; +Dox, treated with 1 µg/ml Dox for 48 h or 72 h; +/− Dox, treated with Dox for 48 h or 72 h and then without Dox for 48 h or 72 h. **d** Quantification of shelterin protein (TIN2) and telomere co-localizing foci. The scale bar represents 10 μm. The data are presented as the mean ± SEM of three independent experiments. **P < 0.01, ***P < 0.001 compared with shPwp1-tet-A ESCs without Dox; ^##^*P* < 0.01, ^###^*P* < 0.001 compared with shPwp1-tet-A ESCs +Dox for 48 h

To further study the impact of *Pwp1* depletion on telomere regulation, we assessed telomere length and the telomeric association of shelterin proteins in sh*Pwp1*-tet-A ESCs upon restoration of *Pwp1* expression. First, telomere length was measured using Q-FISH and qPCR. As shown in Fig. 3a, b, telomere length shortened when *Pwp1* levels were reduced for 48 h but was restored to the pretreatment level when *Pwp1* was re-expressed in sh*Pwp1*-tet-A ESCs by removing Dox. However, after 72 h of *Pwp1* depletion, telomere length shortening could not be reversed upon *Pwp1* restoration. Second, the enrichment of exogenous shelterin protein (TIN2) at the telomeres began to show significant reductions after 48 h of Dox treatment. The reduced enrichment of the exogenous shelterin protein (TIN2) could be restored by withdrawing Dox after 48 h but not after 72 h (Fig. 3c, d), consistent with that of the endogenous shelterin protein (POT1b) in sh*Pwp1*-tet-A KI POT1b ESCs (Supplementary Fig. S5a, b). These data suggested that prolonged *Pwp1* depletion resulted in more permanent changes in telomere length and structures.

### Chromosomal association of *Pwp1* correlated with H4K20me3

**Fig. 4:**
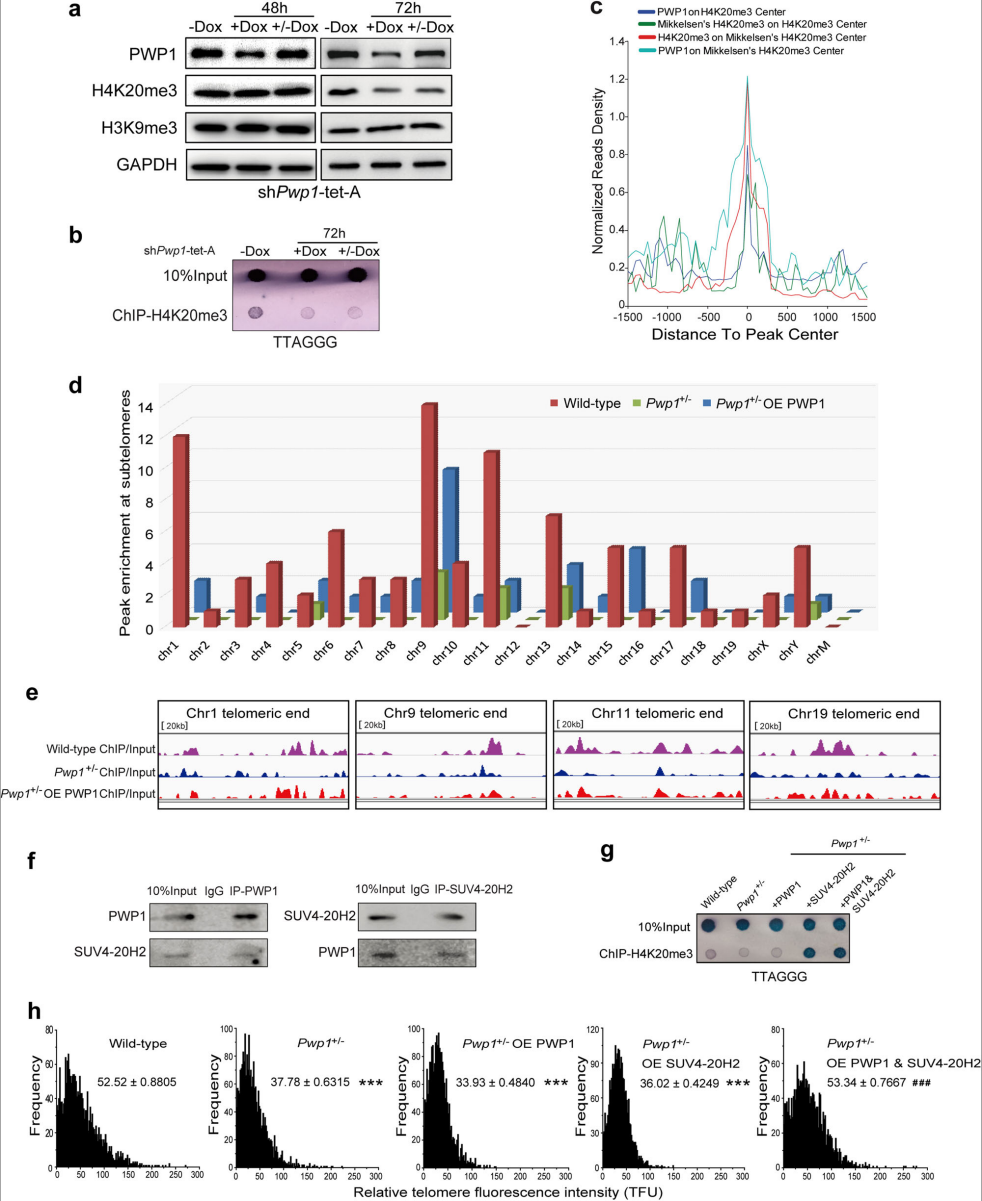
The role of H4K20me3 in the rescue of telomere length in *Pwp1*-depleted ESCs. See also Supplementary Fig. S6. **a** H4K20me3 and H3K9me3 levels in sh*Pwp1*-tet-A ESCs. Cells were treated with 1 µg/ml Dox for 48 h or 72 h. Cell extracts were analyzed by Western blot analysis using the antibodies indicated on the left. −Dox, without Dox treatment; +Dox, treated with Dox for 48 h or 72 h; +/−Dox, treated with Dox for 48 h or 72 h and then without Dox for 48 h or 72 h. **b** H4K20me3 at telomeres. sh*Pwp1*-tet-A ESCs were treated with Dox for 72 h. Chromatin fragments were immunoprecipitated using H4K20me3 antibody, and telomere sequences were detected by dot blot analysis. **c** Distributions of PWP1 sequence reads relative to the H4K20me3 center. Read densities were normalized to reads per kilobases per million reads (RPKM). **d** H4K20me3-devoid islands at subtelomeric regions in wild-type, *Pwp1*^+/−^, and *Pwp1*^+/−^/PWP1-OE ESCs (*P* < 10^−5^ versus random genomic regions). The enrichment (*versus* genome random) of such islands on each subtelomeric region is shown. **e** H4K20me3 enrichment in subtelomeric regions of multiple chromosomes in mouse ESCs. **f** Interaction between PWP1 and SUV4-20H2 proteins. Extracts from ESCs were immunoprecipitated using antibodies against PWP1 or SUV4-20H2 proteins, followed by Western blot analysis using both antibodies. **g** H4K20me3 enrichment at telomeres. Chromatin fragments from wild-type, *Pwp1*^+/−^, PWP1- and SUV4-20H2-overexpressing *Pwp1*^+/−^ ESCs were immunoprecipitated with an antibody against H4K20me3, and telomere sequences were detected on a dot blot. **h** Telomere length in *Pwp1*^+/−^ ESCs. *Pwp1*^+/−^ ESCs were infected with lentiviruses-overexpressing PWP1 or SUV4-20H2. Telomere length was determined by telomere Q-FISH, and the data are shown as TFU. The data are presented as the mean ± SEM of three independent experiments. ***P < 0.001 compared with wild-type ESCs; ^###^*P* < 0.001 compared with Pwp1^+/−^ ESCs

Trimethylations at H3K9 and H4K20 (H3K9me3 and H4K20me3, respectively) are the most important histone modifications in regulating telomere length^11^. To study the effects of *Pwp1* expression on H3K9me3 and H4K20me3 levels, sh*Pwp1*-tet-A ESCs were first treated with Dox for 48 or 72 h and then cultured without Dox for an additional 48 or 72 h. Neither H4K20me3 nor H3K9me3 levels changed significantly after Dox treatment for 48 h (Fig. 4a), despite the fact that telomere length had already decreased at this time. These data suggested that telomere shortening was not a direct result of H4K20me3 reduction. Further, H4K20me3, but not H3K9me3, decreased after Dox treatment for 72 h. Upon Dox withdrawal, the PWP1 protein level recovered to the pretreatment level, but the H4K20me3 level was not restored. ChIP-dot blot assays showed that decreasing *Pwp1* expression also reduced H4K20me3 enrichment in telomeric regions (Fig. 4b). To further explore the changes related to H4K20me3 modifications in the telomeric and subtelomeric regions of chromosomes after *Pwp1* downregulation, ChIP-seq experiments using the H4K20me3 antibody were performed using wild-type, *Pwp1*^*+/−*^, and *Pwp1*^+/−^/PWP1-overexpression (OE) ESCs. As shown in Fig. 4c, H4K20me3 genome enrichment profiles were similar to those of PWP1. This result was consistent with the data reported by Mikkelsen et al.^40^ regarding H4K20me3 in ESCs. Compared with wild-type ESCs, H4K20me3 enrichment was significantly decreased in the subtelomeric regions of *Pwp1*^+/−^ cells and mostly maintained in those of *Pwp1*^+/−^/PWP1-OE cells (Fig. 4d). Specifically, H4K20me3 levels were substantially reduced at subtelomeric regions of Chr1, Chr9, Chr11, and Chr19 in *Pwp1*^+/−^ cells. These changes were not reversed upon subsequent PWP1 overexpression (Fig. 4e). Thus, *Pwp1* depletion-induced changes in chromosomal H4K20me3 modification, especially in the telomeric and subtelomeric regions.

### Restoration of telomere length following *Pwp1* depletion required H4K20me3 catalyzed by SUV4-20H2

Prolonged *Pwp1* depletion led to decreases of both telomere length and H3K20me3 level that were not rescued by subsequent PWP1 overexpression, suggesting that H4K20me3 level was important for the rescue of telomere shortening following *Pwp1* depletion. In mouse ESCs, PWP1 had nuclear localization pattern that was remarkably similar to that of SUV4-20H2, the trimethyltransferase for H4K20me3 (Supplementary Fig. S6a). As shown in Fig. 4f, PWP1 and SUV4-20H2 associated with each other in reciprocal co-IP assays (Fig. 4f and Supplementary Fig. S6b), and *Pwp1* depletion resulted in reduced *Suv4-20h2* expression (Supplementary Fig. S6e). Moreover, the depletion of both *Pwp1* and *Suv4-20h2* led to a shortened telomere length, same as the *Pwp1* depletion (Supplementary Fig. S6c, d). We thought depletion of *Pwp1* had played the leading role on telomere length in the *Pwp1*&*Suv4-20h2* KD ESCs. Next, PWP1 and SUV4-20H2 were overexpressed either individually or in combination in *Pwp1*^+/−^ cells (Supplementary Fig. S6e). H4K20me3 enrichment in telomeric regions was significantly increased in *Pwp1*^+/−^/PWP1 & SUV4-20H2-OE cells (Fig. 4g). The restoration of telomere length required the expression of both PWP1 and SUV4-20H2 proteins (Fig. 4h and Supplementary Fig. S6f). Moreover, an HA-tagged methyltransferase-deficient SUV4-20H2 fragment without the SET domain (HA-SUV4-20H2 DeSET) did not restore the telomere length (Supplementary Fig. S6g, h). Together, these results indicated that H4K20me3 was critical for the regulation of telomere homeostasis by *Pwp1*.

### The second WD40 domain of PWP1 is important for its telomeric functions

**Fig. 5:**
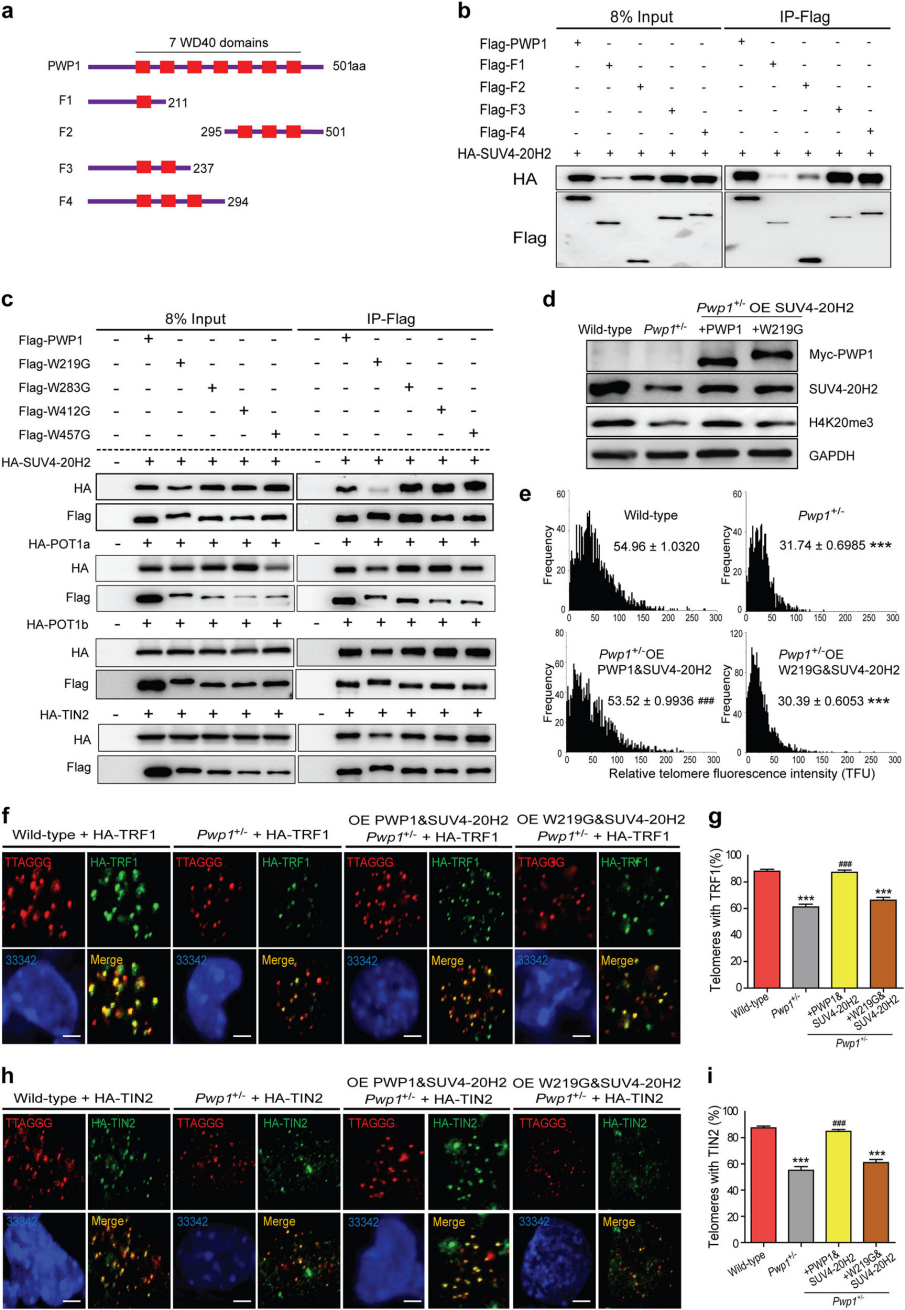
The second WD40 domain of PWP1 plays key roles in stabilizing H4K20 methylation. See also Supplementary Fig. S7. **a** Schematic representation of constructs expressing PWP1 fragments. **b** Interaction between SUV4-20H2 and PWP1 fragments. 293FT cells were cotransfected with plasmids expressing SUV4-20H2 and fragments of PWP1 for 48 h. Cell extracts were immunoprecipitated using an anti-Flag antibody, followed by Western blot analysis using an HA antibody. **c** Binding of SUV4-20H2 and shelterin proteins, POT1a, POT1b, and TIN2, to full-length PWP1 with mutations in WD40 repeats. Plasmids expressing various proteins were co-transfected into 293FT cells for 48 h. Cell extracts were immunoprecipitated using an anti-Flag antibody, followed by Western blotting using an HA antibody. **d** Role of the second WD40 domain of PWP1 in the regulation of SUV4-20H2 expression and cellular H4K20me3 in ESCs. Western blot analysis was performed using ESCs of wild-type, *Pwp1*^+/−^, *Pwp1*^+/−^/SUV4-20H2-OE ESCs transfected with wild-type PWP1 or PWP1(W219G). **e** Relative telomere lengths in wild-type ESCs, *Pwp1*^+/−^ ESCs*, Pwp1*^+/−^ ESCs over-expressing SUV4-20H2 and PWP1 or PWP1(W219G). The telomere lengths were determined by telomere Q-FISH analysis, and the data are shown as TFU. **f**–**i** Telomeric recruitment of shelterin in wild-type ESCs, *Pwp1*^+/−^ ESCs over-expressing SUV4-20H2 and PWP1 or PWP1(W219G). **f**, **h** Representative IF-FISH images. Cells were stained for telomeres (TTAGGG; red), shelterin (TRF1 (**f**) or TIN2 (**h**); green), and nuclei (Hoechst 33342; blue). **g**, **i** Quantifications of (**f**) and (**h**), respectively. The graph shows shelterin proteins (TRF1 or TIN2) and telomere co-localizing foci. The scale bar represents 10 μm. The data are presented as the mean ± SEM of three independent experiments. ***P < 0.001 compared with wild-type ESCs; ^###^*P* < 0.001 compared with Pwp1^+/−^ ESCs

The PWP1 protein contains multiple WD40 repeats. To study the roles of these domains in regulating telomeric functions by PWP1, deletions and point mutations that specifically target its WD40 domains were created^41–43^ (Fig. 5a and Supplementary Fig. S7a–b). As shown in Fig. 5a, we generated four PWP1 deletions: F1 contained the first 211 amino acids containing with one WD40 domain; F2 was a C-terminal fragment containing the last three WD40 repeats; and F3 and F4 were two N-terminal fragments of 237 and 294 amino acids, containing with two and three WD40 domains, respectively. Co-IP of these overexpressed protein fragments in 293FT cells showed that SUV4-20H2 interacted more strongly with F3 and F4 fragments than with F1 and F2 fragments (Fig. 5b), indicating that the second and the third WD40 domains of PWP1, especially the second WD40 domain, played an important role in the interaction between PWP1 and SUV4-20H2. On the other hand, the fragments that contained multiple WD40 domains, F2 and F4, had strong bindings with shelterin (Supplementary Fig. S7c–e). To extend these findings, the interactions between SUV4-20H2 and full-length PWP1 proteins with point mutations at the conserved tryptophan residues (W to G) in WD40 domains were evaluated in 293FT cells. Some of these mutations caused a slight increase in the apparent protein sizes. As shown in Fig. 5c, the interaction with SUV4-20H2 was severely attenuated by the W219G mutation in the second WD40 domain of PWP1 protein, PWP1(W219G). On the other hand, the interaction between PWP1 and shelterin proteins was only marginally affected by the same mutation. Our results suggested that the second WD40 domain of PWP1 protein was critical for the specific association between PWP1 and SUV4-20H2.

Next, we determined the role of the second WD domain of PWP1 protein in rescuing H4K20me3 and telomere defects following *Pwp1* depletion. A plasmid expressing wild-type PWP1 protein or PWP1(W219G) was transfected into *Pwp1*^+/−^ ESCs overexpressing SUV4-20H2. Whereas the H4K20me3 level was restored in cells co-expressing SUV4-20H2 and wild-type PWP1, its level remained lower in cells expressing PWP1(W219G) (Fig. 5d). In addition, overexpression of PWP1(W219G) together with SUV4-20H2 could not restore the telomere length to the same extent as wild-type PWP1 (Fig. 5e). Finally, enrichment of shelterin proteins in telomeric regions was evaluated. The enrichment of shelterin proteins (TRF1 and TIN2) in the telomeric regions of *Pwp1*^+/−^/PWP1(W219G) + SUV4-20H2-OE cells did not recover, compared with those in wild-type ESCs (*Pwp1*^+/+^) and *Pwp1*^+/−^/ PWP1 + SUV4-20H2-OE cells (Fig. 5f–i). Together, these results indicated that WD40 repeats of the PWP1 protein were involved in protein-protein interaction with SUV4-20H2 and shelterin, and the second WD40 domain was especially important for its interaction with SUV4-20H2. As a result, this domain was critical for the involvement of PWP1 in the regulation of telomere length and the enrichment of shelterin proteins in telomeric regions.

### *PWP1* is involved in telomere length regulation in human cells

**Fig. 6:**
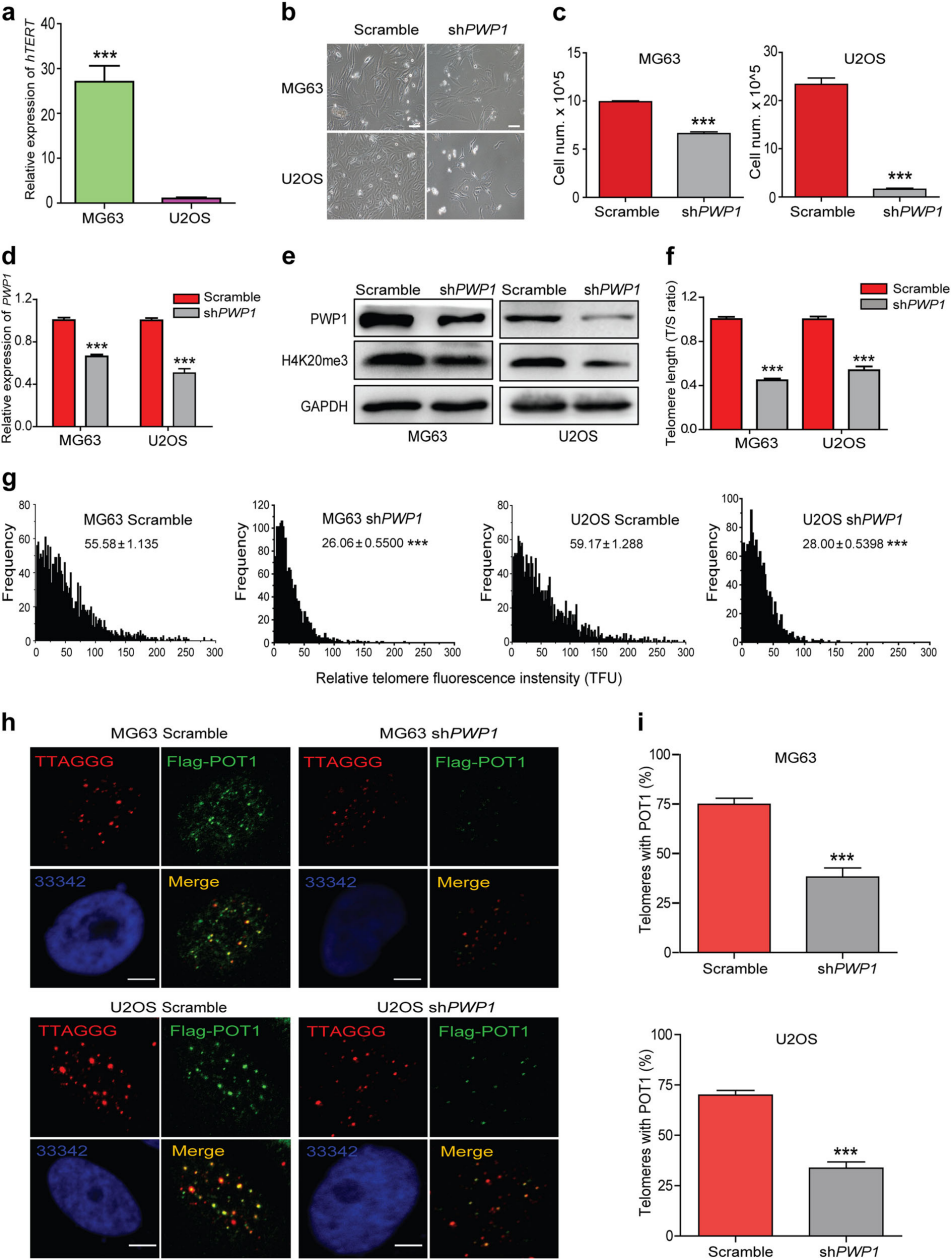
*PWP1* is involved in the regulation of telomere length in human cells. See also Supplementary Fig. S8. **a** *hTERT* expression in human cell lines. *hTERT* mRNA levels in MG63 and U2OS cells were determined by RT-qPCR analysis. **b**, **c** Effects of *PWP1* knockdown in human cell lines. **b** Representative images and (**c**) proliferation of 10^5^ MG63 and U2OS cells. **d**, **e** Knockdown of *PWP1* expression (mRNA (**d**) and protein (**e**) levels) in MG63 and U2OS cells. *PWP1* expression was determined by RT-qPCR analysis, and Western blotting was performed using antibodies against PWP1 protein and H4K20me3. **f**, **g** Changes of telomere length upon *PWP1* knockdown in MG63 and U2OS cells. **f** Relative telomere lengths are shown as the T/S ratio as determined by qPCR. **g** A distribution diagram of relative telomere length. The data are shown as TFU determined by telomere Q-FISH. **h**, **i** Effect of *PWP1* knockdown on Pot1 binding to telomeres. **h** Representative IF-FISH images of MG63 and U2OS cells following *PWP1* knockdown. Cells were stained for telomeres (TTAGGG; red), shelterin (POT1; green), and nuclei (Hoechst 33342; blue). **i** Quantification of POT1 and telomere co-localizing foci. The scale bar represents 10 μm. The data are presented as the mean ± SEM of three independent experiments. ***P < 0.001

To determine whether *PWP1* regulated telomere length/structure in human cells, we performed *PWP1* knockdown in human osteosarcoma cells, MG63 and U2OS, using an shRNA targeting *PWP1* (sh*PWP1*). MG63 and U2OS cells were telomerase-positive and -negative cell lines, respectively (Fig. 6a). Cells were infected with lentiviruses, and puromycin-resistant cells were selected. As shown in Fig. 6b, c, following *PWP1* depletion, both cell lines exhibited reduced cell proliferation capacity, with U2OS cells being affected more substantially. Overall, H4K20me3 levels were decreased in *PWP1*-knockdown cells compared with those in control cells (Fig. 6d, e). Because human cells have shorter telomeres (5–15 kb) than mouse cells (50-150 kb), we conducted TRF analysis to determine telomere length in human cells (Supplementary Fig. S8a). As shown in Fig. 6f, g, telomere shortening was apparent after *PWP1* depletion, similar to that observed in mouse ESCs. We then investigated whether *PWP1* depletion could affect shelterin binding at telomeres in these two cell lines. Upon *PWP1* knockdown, the binding of exogenous shelterin protein (POT1) to telomeres was markedly reduced in MG63 and U2OS cells (Fig. 6h, i). Endogenous shelterin protein (TIN2) also decreased significantly in U2OS cells upon *PWP1* depletion (Supplementary Fig. S8b, c). Together, these data showed that *PWP1* depletion in human cells resulted in reduction in H4K20me3 levels and shelterin assembly in human cells, leading to shortened telomeres.

## Discussion

Compared with somatic cells, ESCs have longer telomeres, which are critical for maintaining their unlimited proliferation and multipotent differentiation capacities. However, mechanisms underlying telomere homeostasis in ESCs remains unclear. Telomeres gradually shorten as ESCs lose telomerase activity. Here, our results showed that *Pwp1* knockdown resulted in a rapid decrease in telomere length, in contrast to what was observed after the loss of telomerase activity. *Pwp1* downregulation did not cause the downregulation of telomerase gene expression or induce changes of telomerase activity or stability. *Zscan4* is an important molecule in the ALT-dependent (telomerase-independent) telomere maintenance mechanism. It has been shown that *Zscan4* silencing leads to shortened telomere length, increases T-SCE, and stalls cell proliferation after 7–8 divisions^16^. Our data showed that, although *Zscan4* decreased upon *Pwp1* depletion, *Zscan4* knockdown did not result in rapid telomere shortening or inhibition of cell proliferation within 48 h. Moreover, ZSCAN4 overexpression did not restore the shortened telomere length in *Pwp1*^+/−^ ESCs. Therefore, *Pwp1* depletion-induced telomere shorten did not involve reduced telomerase activity or *Zscan4*-dependent ALT mechanism.

Studies have shown that the shelterin complex plays a very important role in telomere protection. Shelterin maintains telomeric 3′ overhang, forms and protects telomere t-loop structure^44^. Formation of the 3′ overhang at mouse telomeres involves an intricate set of steps that are controlled by POT1b and TRF2^45^. In human cells, the TPP1-POT1 unit can cap telomere ends and regulate telomerase access to telomere ssDNA^46,47^. Abnormal expression, mutation or depletion of different shelterin components have different effects on telomere length and stability. For example, heterozygous mutation of TPP1 causes telomere shortening and the development of telomere-related diseases^48^. Removing TRF1 leads to very frequent replication fork stalling in the telomeric region^49,50^. Moreover, combinational depletion of several shelterin components has more dramatic effects on telomere structures than single depletion of one protein^51^. Our results showed that PWP1 associated with shelterin proteins (POT1a, POT1b, TPP1, and TIN2). *Pwp1* depletion led to a simultaneous reduction of enrichment of most shelterin proteins at telomeres, which impaired shelterin protection and disturbed telomere homeostasis. Therefore, our results indicated that the telomere shortening in *Pwp1*-deficient cells was triggered by the loss of shelterin enrichment in the telomeric region.

*Pwp1* depletion activated DNA damage signaling in both the telomeric region and the nucleus. PWP1 has the WD40 repeats structure, which endows it with an ability of protein binding and a possibility of participating in different regulatory networks^52,53^. In the telomeric region, depletion of *Pwp1* reduced the shelterin protein enrichment and impaired the shelterin complex protection, in turn aggravating the DNA damage. In *Pwp1*-deficient ESCs, DNA damage was also aggravated in the nucleus, possibly because of other impaired PWP1 binding relationships. The DNA damage has many factors and the WD40 repeats protein could affect DNA damage in the nucleus^54,55^. PWP1 was widely expressed and present throughout the nucleus, suggesting that it played roles at and outside telomeres. Therefore, PWP1, which indeed had locations at the telomeric and subtelomeric regions and bindings with the shelterin complex, could prevent DNA damage at telomeres, and it might play roles in DNA damage of non-telomeric region.

Studies from de Lange laboratory have shown that *Tin2* heterozygous deletion causes telomere shortening and that its homozygous deletion is embryonic lethal in mice. This telomere length shortening occurs despite the presence of sufficient telomerase activity^35^. This finding is similar to ours regarding *Pwp1*-knockout mice and ESCs. Furthermore, telomere lengthening occurs when germ cells develop into haploid sperm cells in mammals. Our previous studies showed that telomerase was highly expressed in spermatids of mouse testicular tissues^56^. In this study, we showed that the *Pwp1* was highly expressed in testicular tissues and that *Pwp1* heterozygous-knockout mice had significantly reduced reproductive capability, suggesting that *Pwp1* played a role in telomere lengthening during spermatogenesis.

Histone modifications are critical for telomere maintenance and regulation. Liu et al. has shown that RIF1 interacted with the H3K9 methylation complex to stabilize the level of H3K9me3 modification and negatively regulate ZSCAN4 expression to achieve the dynamic regulation of telomere length^57^. Recently, Konishi et al. also found that the interaction between TRF2 and core histones was important for the stabilization of chromosome ends. Mutations in the GAR domain of TRF2 caused a loss of telomere protection and resulted in rapid telomere shortening^58^. In our current study, the co-IP data showed that PWP1 interacted with SUV4-20H2, a methyltransferase for H4K20 trimethylation. ChIP-seq data showed that genome-wide PWP1 binding sites matched H4K20me3 sites. Lastly, both H4K20me3 level and telomere length were reduced upon *Pwp1* depletion. Consistently, it was previously reported that H4K20me3 enrichment in telomeric and subtelomeric regions was significantly decreased in *Terc-*knockout mice with shortened telomeres^27^. Our data also showed that PWP1 overexpression in ESCs with *Pwp1* knockdown or heterozygous knockout could not restore telomere length or the level of H4K20me3. However, overexpression of both PWP1 and SUV4-20H2 restored the H4K20me3 level and telomere length and increased localization of shelterin complexes to telomeric regions, indicating that H4K20me3 is crucial in *Pwp1*-mediated telomere maintenance.

**Fig. 7:**
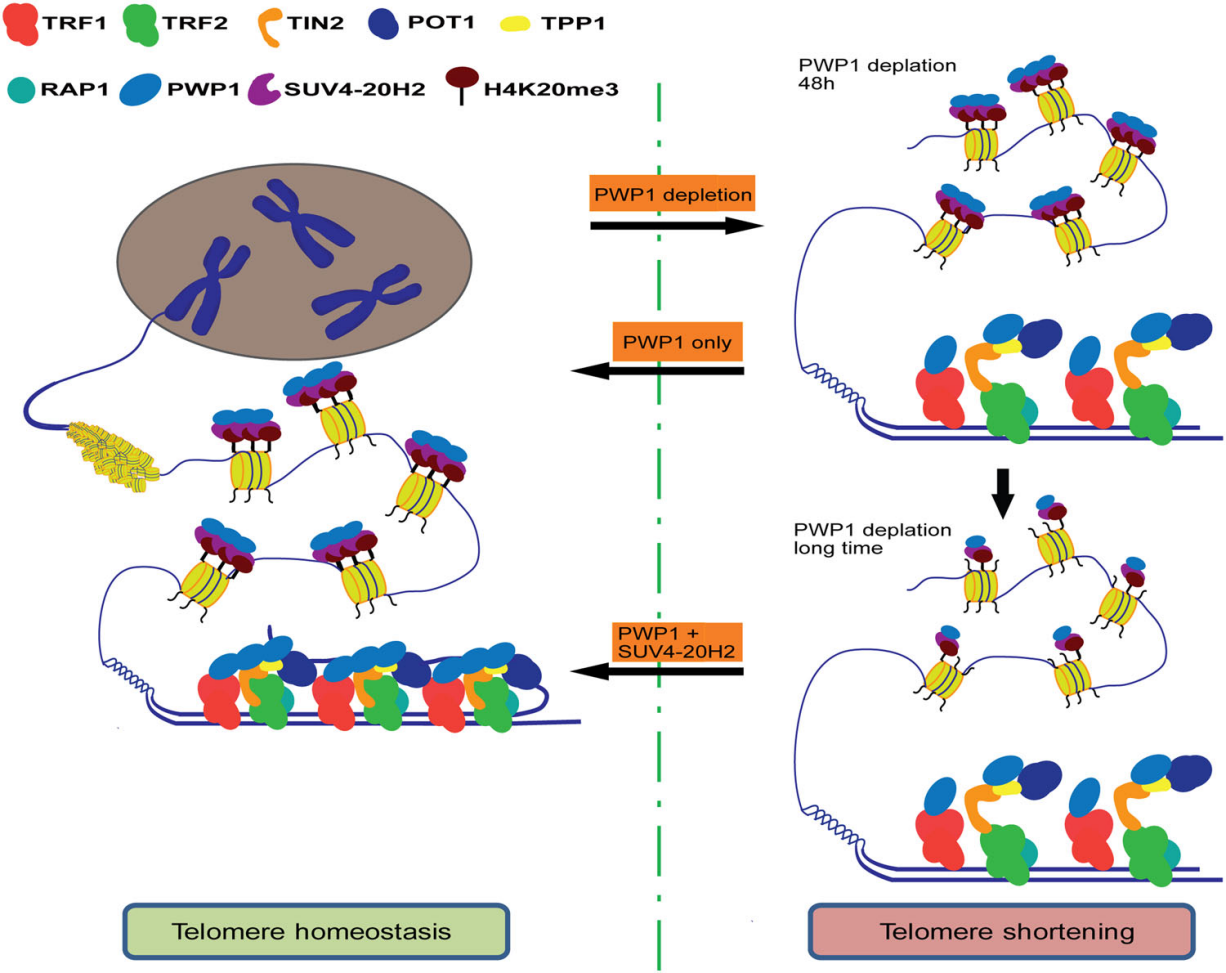
A model of *Pwp1’*s roles in regulating telomere length and function. Left: In wild-type cells, PWP1 binds with and stabilizes shelterin protein complexes and regulates the overall level of H4K20me3 to maintain telomere homeostasis. Right: Within 48 h, the absence of *Pwp1* resulted in the reduction of shelterin enrichment in telomeres and rapid telomere shortening. The rescue of telomere length in *Pwp1*-deficient cells depended on PWP1 expression alone. After a prolonged *Pwp1* depletion, the H4K20me3 enrichment was reduced in the subtelomeric and telomeric regions of chromosomes. The rescue of telomere length in *Pwp1*-deficient cells by PWP1 overexpression also depended on SUV4-20H2 co-expression and increased H4K20me3

PWP1 has seven WD40 repeats^41^. These domains have important functions in protein-protein interactions. Our mutagenesis study showed that the second WD40 repeat of PWP1 was important for its interaction with SUV4-20H2. Overexpression of PWP1^W219G^ and SUV4-20H2 in *Pwp1*^+/−^ ESCs failed to restore H4K20me3 levels, telomere length, or the localization of the shelterin complex to telomeric regions. Hence, the interaction between the second WD40 repeat of PWP1 protein and SUV4-20H2 was likely important not only for restoring the H4K20me3 level, but also for shelterin complex stabilization and telomere maintenance. While shelterin instability might be the primary reasons for the rapid telomere shortening induced by *Pwp1* deficiency, the restoration of telomere length by *Pwp1* could not occur without an adequate level of H4K20me3 (Fig. 7). In conclusion, our study revealed a new telomere regulatory mechanism involving the chromatin-binding protein PWP1 as a bridge that facilitated both the recruitment of telomere end protective proteins and histone modifications at telomeric regions.

## Materials and methods

### Cell culture

Mouse ESCs (E14.1) were cultured on plates coated with 0.1% gelatin (Millipore) in DMEM (Gibco) supplemented with 15% (v/v) fetal bovine serum (FBS, Gibco), 2 mM GlutaMAX (Gibco), 1 mM sodium pyruvate (Gibco), 0.1 mM non-essential amino acids (Gibco), 1000 units/ml leukemia inhibitory factor (LIF, Millipore), and 0.18% β-mercaptoethanol (Sigma). Medium was changed daily, and cells were routinely passaged every two days. Additionally, 293FT, MG63, and U2OS cells were cultured in DMEM containing 10% FBS.

### Gene knockdown, knockout, knock in or overexpression

In mouse ESCs, for *Pwp1* knockdown, an shRNA targeting *Pwp1* mRNA was designed (sh*Pwp1*-tet-A, Table S1) and cloned into the lentivirus vector pLKO-Tet-On (a gift from Zhang Xiaoqing Lab, Tongji University, China). For *Pwp1* knockout, a gRNA targeting the major open reading frame of *Pwp1* was designed (*Pwp1* gRNA, Table S1) and cloned as previously described^59^. For HA-POT1b knock in, a gRNA was designed (HA-*Pot1b* gRNA, Table S1) through the website Optimized CRISPR Design (http://crispr.mit.edu/). For protein overexpression, cDNAs were cloned into the vector FUGW (Addgene), and plasmids were electroporated into E14.1 mESCs using Gene Pulser X Cell System (Bio-Rad).

To knockdown *PWP1* in human cells, an shRNA targeting *PWP1* mRNA was designed (sh*PWP1*-A, Table S1) and cloned into the lentivirus vector pLKO.1 (Addgene). Human shelterin plasmids (gifts from Dr. Mao Zhiyong Lab, Tongji University, China) were electroporated into cells using Gene Pulser X Cell System (Bio-Rad).

### Reverse transcription and quantitative PCR (RT-qPCR)

Total RNAs were isolated using TRIzol (Invitrogen) and reverse transcribed to generate cDNAs using a Prime-Script RT reagent kit (TaKaRa). cDNAs were amplified with the SYBR Premix Ex Taq (TaKaRa). The primer sequences used for qPCR are listed in Table S1.

### Telomere measurement by qPCR

Genomic DNA was prepared using the TIANamp Genomic DNA Kit (DP304) (TIANGEN). Average telomere length was measured from total genomic DNA using a modified qPCR assay^60,61^. Equal amounts of DNA were used for each reaction. The primers used in the PCR reactions were telomeric primers and primers for the reference control gene (Mouse 36B4 single copy gene and human hBg single copy gene). The primer sequences are listed in Table S1.

### Western blot and co-IP

Cells were lysed in RIPA buffer containing a protease inhibitor cocktail (Roche). Total protein from cell extracts were resolved by 10–12% Bis-Tris SDS-PAGE and transferred to polyvinylidene difluoride (PVDF) membranes (Millipore). Blots were blocked by incubation in 3% BSA in TBST at RT for 1 h, probed with various primary antibodies overnight at 4 °C in TBST and with secondary antibodies at RT for 1 h. Signals were visualized using enhanced chemiluminescence (ECL Hakata, ImageQuant LAS 4000 mini).

Co-IP was performed as previously described^62^. For the endo-co-IP, cell lysates were incubated with antibodies or control normal IgG overnight at 4 °C. Then, a mixture of Ezview Red Protein A affinity gel (Sigma) and Ezview Red Protein G affinity gel (Sigma) (1:1) was added to the antibody-containing lysates for 2 h. For the exogenous co-IP, cell lysates were immunoprecipitated with anti-FLAG or anti-HA M2 magnetic beads (Sigma) for 4 h at 4 °C. Bead-protein mixtures were subjected to Western blotting after washing. The antibodies used for western blot and co-IP are listed in Table S2.

### Telomere quantitative fluorescence in situ hybridization (Q-FISH)

Cells were incubated with 0.2 μg/ml colcemid (Sigma) for 4 h to enrich cells at metaphase. Metaphase-enriched cells were harvested by trypsinization, resuspended in 75 mM KCl at 37 °C for 5 min, fixed with methanol/glacial acetic acid (3:1) at room temperature (RT) for 1 h, and spread onto slides. Telomere FISH and quantification were performed as described previously^63^ except that a fluorescein isothiocyanate (Cy3)-labeled (TAAGGG)_3_ peptide nucleic acid (PNA) probe (Panagene) was used in this study. Telomeres were denatured at 80 °C for 10 min and hybridized with the telomere-specific PNA probe (0.5 mg/ml). Chromosomes were counterstained with 0.5 mg/ml DAPI.

### Immunofluorescence-telomere FISH (IF-FISH)

The IF-FISH method was performed based on an established protocol^64^. Cells were grown on gelatin-treated coverslips, fixed with 2% paraformaldehyde for 10 min, and then blocked with blocking solution (1 mg/ml BSA, 3% goat serum, 0.1% Triton X-100, and 1 mM EDTA, pH 8.0) for 1 h at RT, followed by incubation with primary antibodies in blocking solution at 4 °C overnight. The coverslips were then incubated for 1 h at RT with secondary antibodies and again fixed with 2% paraformaldehyde for 5 min FISH was performed as described earlier. Cells were stained with 0.5 mg/ml Hoechst 33342 for 10 min at RT.

### RNA immunoprecipitation (RIP)

RIP was performed as previously described^65^. Briefly, 5 × 10^6^ cells were lysed in RIP buffer (10mM HEPES pH 7.0, 5mM MgCl_2_, 100mM KCl, 1mM Dithiothrectol, and 0.5% NP-40) for 30 min at 4 °C. And 4 μg of antibody was used in each RIP assay to pull down the RNAs associated with the corresponding proteins. Finally, the co-immunoprecipitated RNAs were extracted, reverse transcribed to generate cDNAs and then analyzed by RT-qPCR.

### Chromatin immunoprecipitation (ChIP)

ChIP assays were performed using the Simple ChIP Plus Enzymatic Chromatin IP Kit (Magnetic Beads) (#9005, Cell Signaling Technology). Briefly, 10^7^ cells were crosslinked with 1% formaldehyde for 10 min at RT, and formaldehyde was quenched with 0.125 M glycine. Samples were lysed and sonicated to generate fragments of 500–750 bp. The fragments were immunoprecipitated in ChIP buffer with ChIP-Grade Protein G magnetic beads coupled to specific antibodies and eluted in ChIP Elution Buffer, and the cross-linking was reversed overnight at 65 °C. Samples were treated with Proteinase K and RNase A and extracted with DNA purification columns.

### ChIP sequencing (ChIP-seq)

H4K20me3 ChIP-seq reads were processed to filter out reads with low-quality using cutadapt (version 1.8.3) and trimmomatic (version 0.36)^66,67^. The remaining reads were mapped to mm10 (GRCm38) using Bowtie2 with default parameters^68^. Only uniquely aligned reads were used for subsequent analysis. Peaks were detected using the MACS2 with parameters -p and -broad-cutoff set to 10-5 (version 2.1.0)^69^. The R package-ChIPseeker was used to annotate the identified peaks and visualize differential H4K20me3 enrichment following *Pwp1* depletion and restoration^70^. The R language (https://www.r-project.org/) was used to perform hypothesis testing. PWP1 and Mikkelsen’s H4K20me3 ChIP data were downloaded from the Gene Expression Omnibus (GEO) database (GSE59389, GSM307622). The ChIP-seq data have been deposited in the European Bioinformatics Institute Database Array Express (https://www.ebi.ac.uk/arrayexpress/) under accession number E-MTAB-5985.

### Dot blot

DNA samples were denatured in 0.1 M Tris-HCl, pH 8.5, and 0.1 M NaCl for 10 min at 95 °C, mixed with cold 2 M NH_4_Ac (equal volume), and spotted on a nitrocellulose membrane. The membrane was dried at 65 °C for 5 min and crosslinked for 90 s. A DIG-labeled probe was added to the hybridization buffer and incubated with the membrane overnight at 37 °C. The probes were performed as described previously^71^. The membrane was developed using the DIG Nucleic Acid Detection Kit (Catalog No. 11175041910, Roche).

### TRAP assay

TRAP assays were performed using the TRAPeze® Telomerase Detection Kit (S7700, Millipore). Cells were resuspended in 1× CHAPS lysis buffer freshly supplemented with RNase inhibitors, incubated on ice for 30 min, and spun in a microcentrifuge at 12,000 × *g* for 20 min at 4 °C, followed by PCR analysis. The PCR products were run on a 10% or 12.5% non-denaturing PAGE gel and stained with ethidium bromide.

### Comet assay

Comet assays were performed using Trevigen Comet Assay® Kit. Cells were mixed in Comet LMAgarose. The mixture (50 µl) was spread on the surface of Comet Slides. The slides were incubated at 4 °C for 30 min, immersed in lysis solution for 30 min at 4 °C, soaked in alkaline unwinding solution for 30 min at RT, and then electrophoresed in the Comet Assay® ES system and stained with SYBR Green I.

### Telomere restriction fragment (TRF) analysis

The TRF analysis was performed based on an established protocol^72^. Southern blot assays were performed using the North2South™ Chemiluminescent Hybridization and Detection Kit (17097, Thermo Fisher Scientific). Equal amounts of genomic DNA were used for each reaction.

### Protein structural model

The web server Hhpred (https://toolkit.tuebingen.mpg.de/#/tools/hhpred) was employed to model the protein structures. PDB_mmCIF70 was used as the target database to generate a homology model. The best hits were selected as templates for modeling.

### Animal study

All procedures involving animals were approved by the Laboratory Animal Care Committee of Tongji University under the Guide for the Care and Use of Laboratory Animals (NIH Guide). All mice were maintained in a pathogen-free environment throughout the experiments, and all efforts were made to minimize the number of animals used and their suffering. Production of *Pwp1*^+/−^ mice was conducted as previously described^73^.

### Statistical analysis

The values are presented as the mean ± SEM. Significance was determined using two-tailed Student’s t tests and GraphPad Prism software.

## Supplementary information

Supplementary information.

## Supplementary information

**Supplementary Information** accompanies the paper at (10.1038/s41421-019-0116-8).
