# Supplementary material for: Pwp1 regulates telomere length by stabilizing shelterin complex and maintaining histone H4K20 trimethylation
Source: Cell Discov. 2019 Nov 5;5:47. doi: 10.1038/s41421-019-0116-8 (PMC6868014; doi:10.1038/s41421-019-0116-8)
Supplement: Supplementary file 1 — Supplementary information. [file 41421_2019_116_MOESM1_ESM.pdf]

Supplementary Information

Supplementary Fig.S1

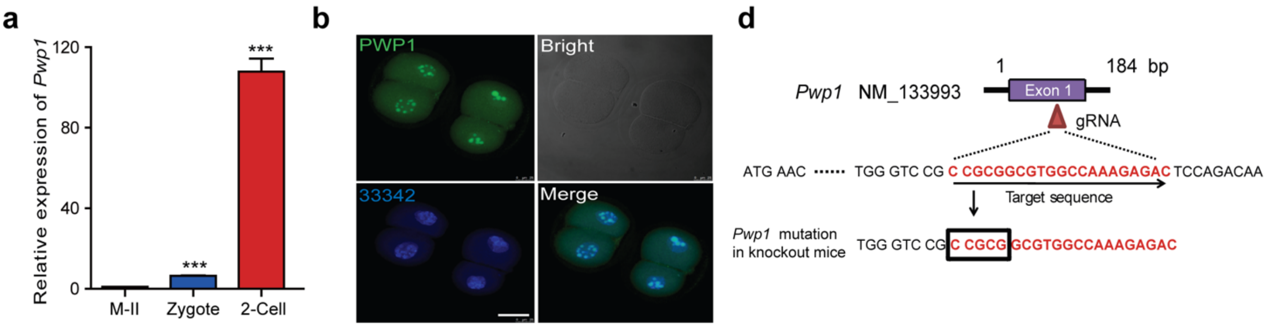

c

	No. transferred embryos	No. transplanted pseudopregnant	No. pregnancy	No. born pups	No. SKO *Pwp1* pups
Control	120	8	6	48	-
Pwp1 gRNA	180	10	2	6	2

e

Wild-type embryos	No. breeding *Pwp1*$^{+/-}$ F&M embryos	*Pwp1*$^{+/-}$ ES cell line	*Pwp1*$^{-/-}$ ES cell line	Wild-type cell line
-	20	6	0	8
5	-	-	-	4

f

No.pickup ES clones	No. *Pwp1*$^{+/-}$ ES clones	No. *Pwp1*$^{-/-}$ ES clones
56	10	0

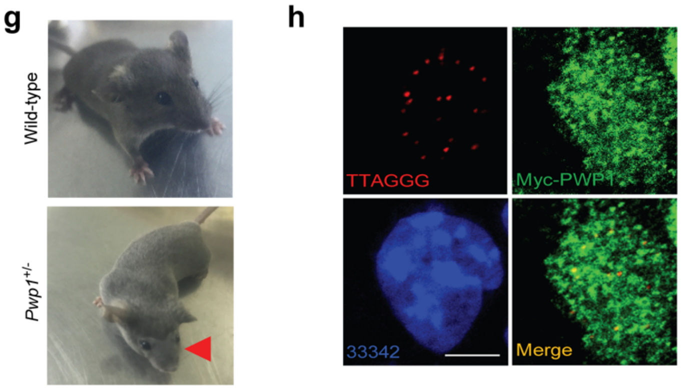

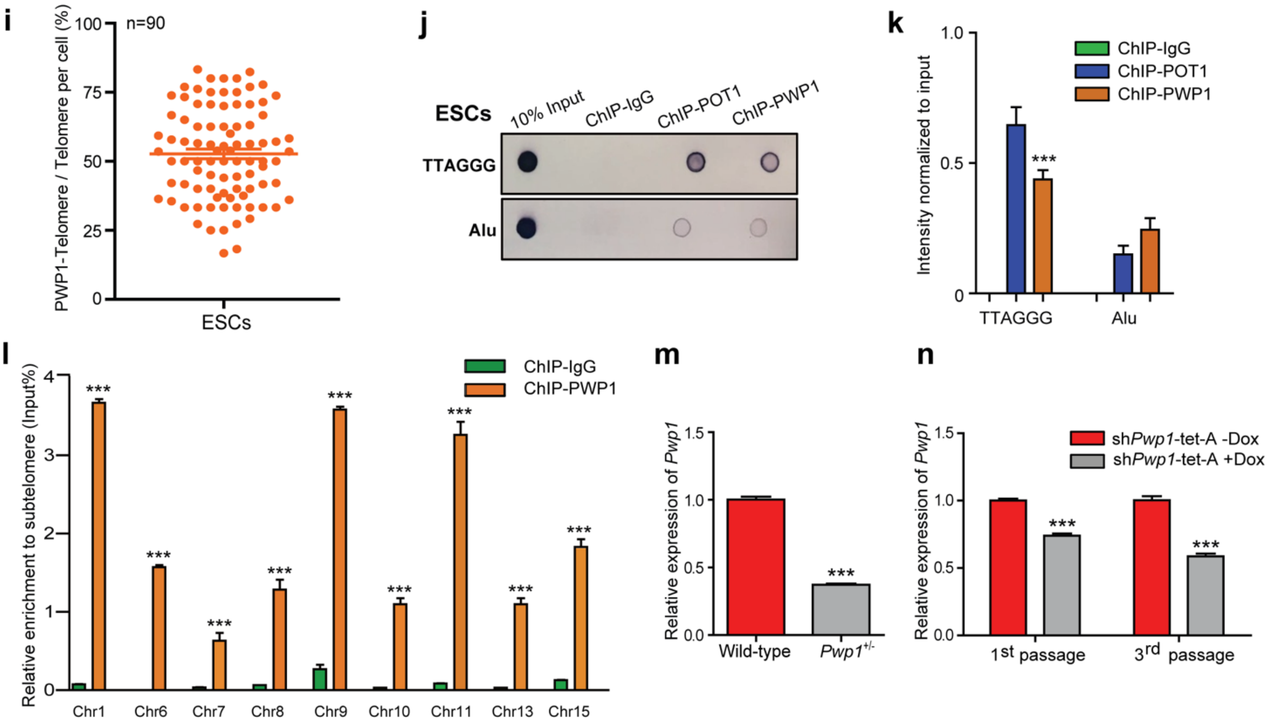

Supplementary Fig. S1: Depletion of *Pwp1* affects mouse embryonic development and mESC telomere length. Related to Fig. 1.

a *Pwp1* expression determined by qPCR analysis in the mouse early embryonic development period (M-II, Zygote, 2-cell).

b IF analysis of PWP1 expression during development at the 2-cell stage. Cells were stained for PWP1 (green) and nuclei (Hoechst 33342; blue). The scale bar represents 50 μm.

c Number of *Pwp1*-knockout pups obtained by CRISPR/Cas9 editing.

d Schematic illustration of the gRNA target region targeting the mouse *Pwp1* gene. The *Pwp1* gene in the knockout line contains a 5-bp deletion in its coding region.

e Number of ES cell lines obtained by the breeding of $Pwp1^{+/-}$ male and female embryos.

f The number of ESC clones obtained by modifying the *Pwp1* gene using CRISPR/Cas9 editing.

g Representative images of wild-type and $Pwp1^{+/-}$ mice. Red triangle, absence of hair.

h IF-FISH analysis of PWP1 localization at mouse ESC telomeres. Cells were stained for telomeres (TTAGGG; red), Myc-PWP1 (Myc; green), and nuclei (Hoechst 33342; blue). The scale bar represents 10 μm.

i Quantification of co-localizing foci of PWP1 and telomeres. The graph shows the percentage of PWP1-Telomere co-localizing foci among the total telomeres foci per cell. The total number of ESCs is 90.

j PWP1 at telomeres. Chromatin fragments were immunoprecipitated using antibodies (positive control, POT1; negative control, IgG) and hybridized on a dot blot with either telomere or control (Alu) probe.

k Quantification of PWP1 ChIP-Dot blot. The results were quantitated with ImageJ software.

l PWP1 ChIP at the subtelomeric loci in ESCs.

m *Pwp1* expression determined by qPCR analysis in wild-type and $Pwp1^{+/-}$ ESCs.

n *Pwp1* expression determined by qPCR analysis in sh*Pwp1*-tet-A ESCs at passages 1 and 3.

*The data are presented as the mean ± SEM of three independent experiments. ***P < 0.001.*

Supplementary Fig.S2

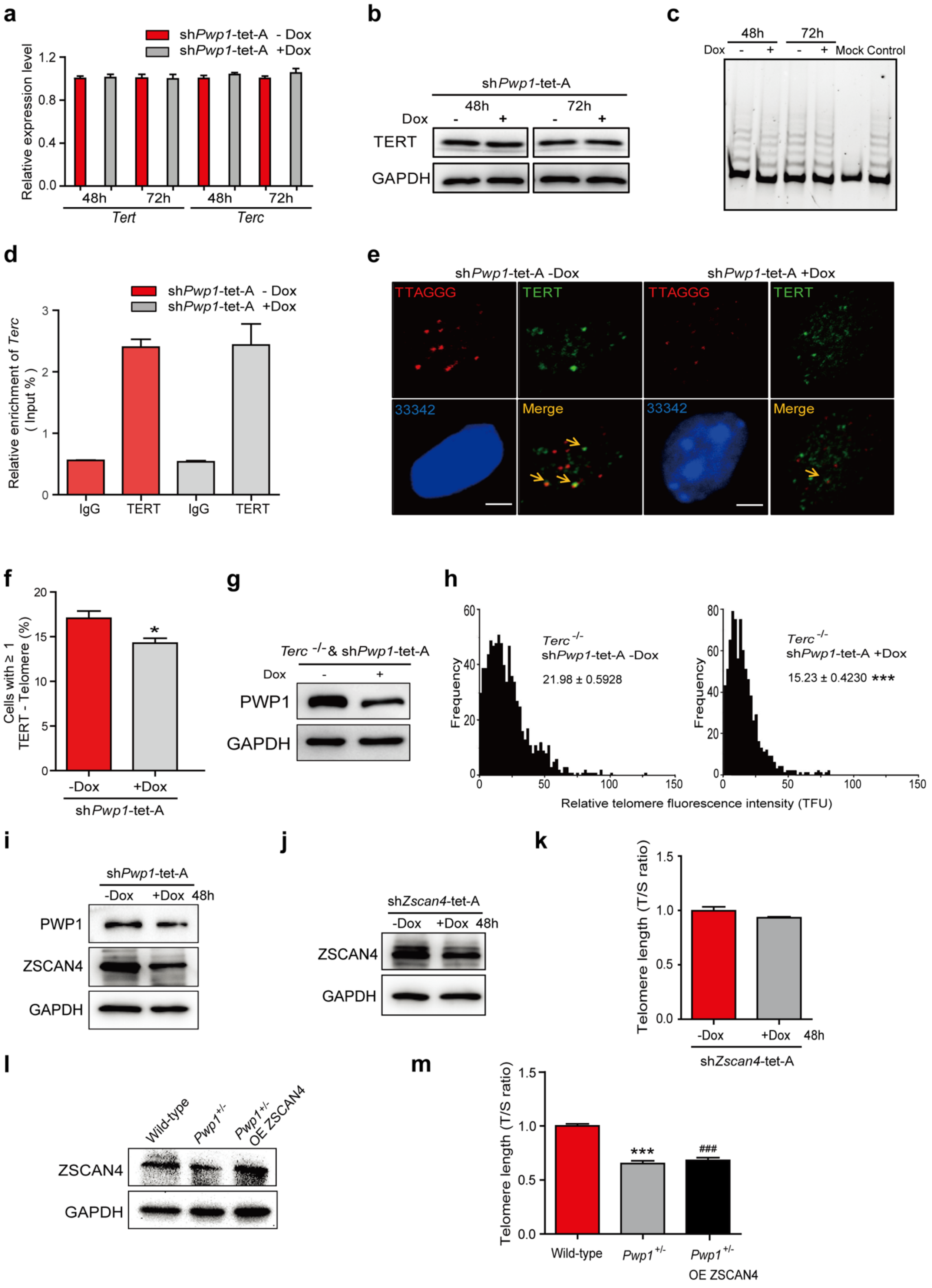

Supplementary Fig. S2: Telomere shortening in *Pwp1*-depleted ESCs is not due to telomerase or *Zscan4*. Related to Fig. 2.

a-b The effects of *Pwp1* knockdown on telomerase expression in sh*Pwp1*-tet-A ESCs. The mRNA levels were determined by qPCR analysis (**a**), and the protein levels were determined by Western blot analysis (**b**).

c The telomerase activities were measured by TRAP assays in sh*Pwp1*-tet-A ESCs. Mock, RNase A-treated cell lysates; Control, telomerase-positive cell lysates.

d *Terc* enrichment for TERT was measured by RIP assays in sh*Pwp1*-tet-A ESCs treating with 1 μg/ml Dox for 48 hours.

e IF-FISH analysis of TERT localization at telomeres. The sh*Pwp1*-tet-A ESCs were treated with Dox for 48 hours. Cells were stained for telomeres (TTAGGG; red), TERT (green), and nuclei (Hoechst 33342; blue). The scale bar represents 10 μm.

f Quantification of TERT and telomere co-localizing foci. The graph shows the percentage of cells presenting one or more TERT and telomere co-localizing foci. $^{*}P < 0.05$.

g PWP1 expression was determined by Western blot analysis in $Terc^{-/-}$ & sh*Pwp1*-tet-A ESCs.

h Relative telomere lengths were determined by QFISH in $Terc^{-/-}$ & sh*Pwp1*-tet-A ESCs. The data are shown as the mean TFU ± SEM of three independent experiments. $^{***}P < 0.001$.

i ZSCAN4 protein levels in *Pwp1*-knockdown cells. ESCs containing sh*Pwp1*-tet-A were treated with 1 μg/ml Dox for 48 hours, and Western blot analysis were performed.

j Knockdown of ZSCAN4 protein levels in ESCs. ESCs containing sh*Zscan4*-tet-A were treated with or without 1 μg/ml Dox for 48 hours. ZSCAN4 protein expression was determined by Western blot analysis.

k Telomere length upon *Zscan4* knockdown. ESCs containing sh*Zscan4*-tet-A were treated with or without 1 μg/ml Dox for 48 hours. Relative telomere length was determined by qPCR analysis.

l ZSCAN4 protein levels in *Pwp1*-deficient ESCs. Wild-type, $Pwp1^{+/-}$, and $Pwp1^{+/-}$/ZSCAN4 overexpressing (OE) ESCs were examined by Western blot analysis using antibody against ZSCAN4.

m Telomere length in wild-type, $Pwp1^{+/-}$, and $Pwp1^{+/-}$/ZSCAN4 OE ESCs. Relative telomere length was determined by qPCR. $^{***}P < 0.001$ compared with wild-type ESCs and $^{###}P < 0.001$ compared with $Pwp1^{+/-}$ ESCs.

The data are presented as the mean ± SEM of three independent experiments.

Supplementary Fig.S3

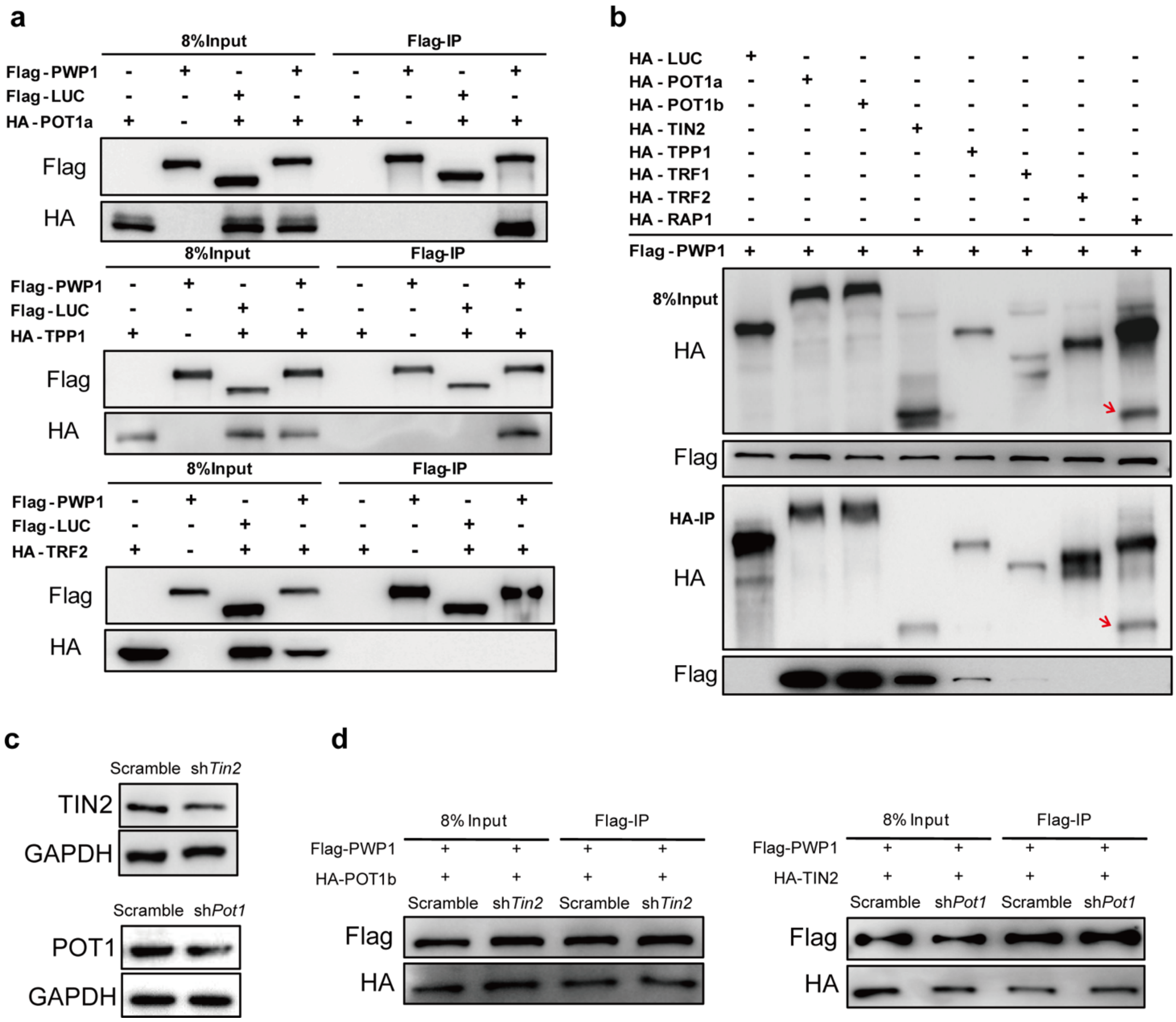

Supplementary Fig. S3: The binding between PWP1 and shelterin. Related to Fig. 2.

a Binding between PWP1 and shelterin. Flag-PWP1 and HA-Shelterin were co-transfected into 293FT cells. Cell extracts were immunoprecipitated with a Flag antibody, and Western blot analysis was performed using an HA antibody.

b Binding between shelterin and PWP1. Flag-PWP1 and HA-Shelterin were co-transfected into 293FT cells. Cell extracts were immunoprecipitated with an HA antibody, and Western blot analysis was performed using a Flag antibody.

c Expression was determined by Western blot analysis in *Tin2* KD and *Pot1* KD ESCs.

d Binding between PWP1 and POT1 or TIN2 in *Tin2 KD*/PWP1& POT1b-OE ESCs or *Pot1* KD/ PWP1&TIN2-OE ESCs. Cell extracts were immunoprecipitated with a Flag antibody, and Western blot analysis was performed using an HA antibody.

Supplementary Fig.S4

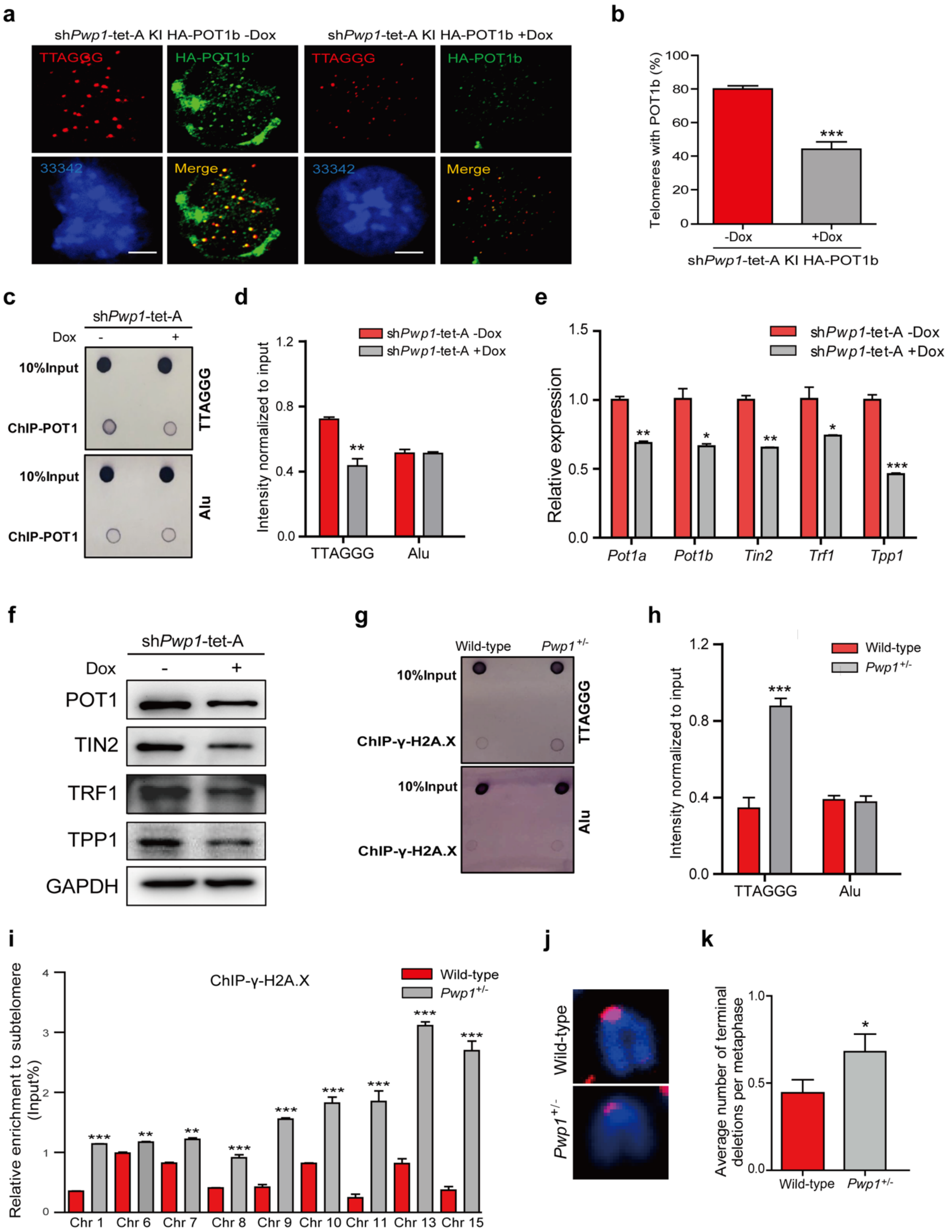

72

Supplementary Fig. S4: The shelterin and DNA damage changes in *Pwp1*-delepted ESCs. Related to Fig. 2.

a Representative images of shelterin protein (POT1b) localization in sh*Pwp1*-tet-A KI HA-POT1b ESCs treating with 1 μg/ml Dox for 48 hours. Cells were stained for telomeres (TTAGGG; red), shelterin (HA-POT1b; green), and nuclei (Hoechst 33342; blue). The scale bar represents 10 μm.

b Quantification of shelterin protein (POT1b) and telomere co-localizing foci.

c Shelterin protein (POT1) at telomeres in sh*Pwp1*-tet-A ESCs treating with 1 μg/ml Dox for 48 hours. Chromatin fragments were immunoprecipitated using the POT1 antibody and hybridized on a dot blot with either telomere or control (Alu) probe.

d Quantification of shelterin protein (POT1) ChIP-Dot blot. The results were quantitated with ImageJ software.

e Expression levels of shelterin determined by qPCR analysis in sh*Pwp1*-tet-A ESCs treating with 1 μg/ml Dox for 48 hours.

f Expression levels of shelterin determined by Western Blot analysis in sh*Pwp1*-tet-A ESCs treating with 1 μg/ml Dox for 48 hours.

g γ-H2A.X at telomeres in wild-type ESCs and $Pwp1^{+/-}$ ESCs. Chromatin fragments were immunoprecipitated using the γ-H2A.X antibody and hybridized on a dot blot with either telomere or control (Alu) probe.

h Quantification of γ-H2A.X ChIP-Dot blot. The results were quantitated with ImageJ software.

i γ-H2A.X ChIP at the subtelomeric loci in wild-type ESCs and $Pwp1^{+/-}$ ESCs.

j Representative images of terminal deletions in wild-type ESCs and $Pwp1^{+/-}$ ESCs. Cells were stained for telomeres (TTAGGG; red) and nuclei (DAPI; blue).

k Quantification of terminal deletions.

*The data are presented as the mean ± SEM of three independent experiments. *P < 0.05, **P < 0.01 and ***P < 0.001*

Supplementary Fig.S5

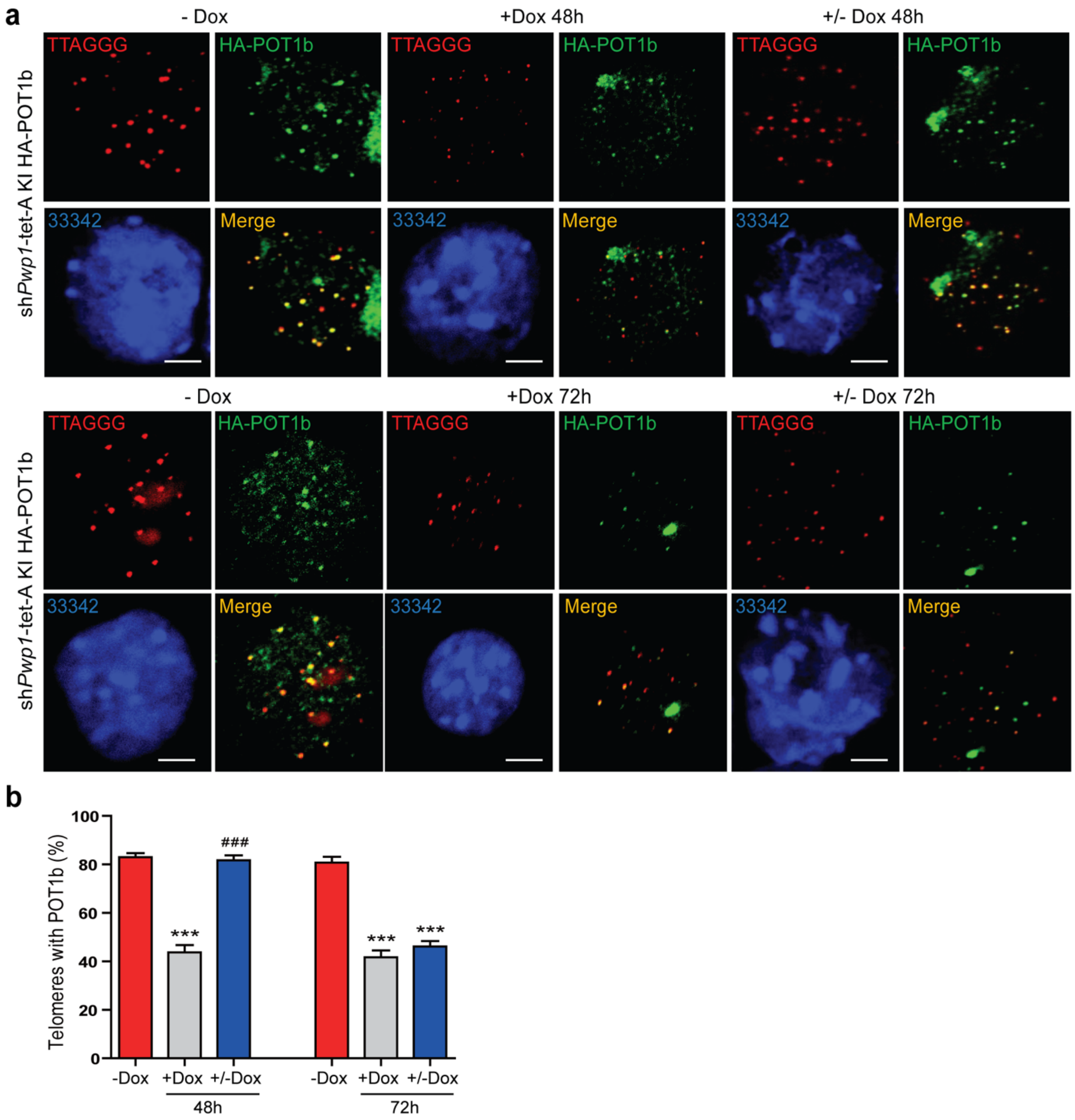

Supplementary Fig. S5: Endogenous shelterin enrichment changes at telomeres. Related to Fig. 3.

a Representative images of shelterin protein (POT1b) localization in sh*Pwp1*-tet-A KI HA-POT1b ESCs. -Dox, without Dox treatment; +Dox, treated with Dox for 48 hours or 72 hours; +/- Dox, treated with Dox for 48 hours or 72 hours and then without Dox for 48 hours or 72 hours. Cells were stained for telomeres (TTAGGG; red), shelterin (HA-POT1b; green), and nuclei (Hoechst 33342; blue). The scale bar represents 10 μm.

b Quantification of shelterin protein (POT1b) and telomere co-localizing foci. The data are presented as the mean ± SEM of three independent experiments. $^{***}P < 0.001$ compared with the sh*Pwp1*-tet-A KI HA-POT1b -Dox ESCs, and $^{\#\#\#}P < 0.001$ compared with the sh*Pwp1*-tet-A KI HA-POT1b +Dox 48-hour ESCs.

Supplementary Fig.S6

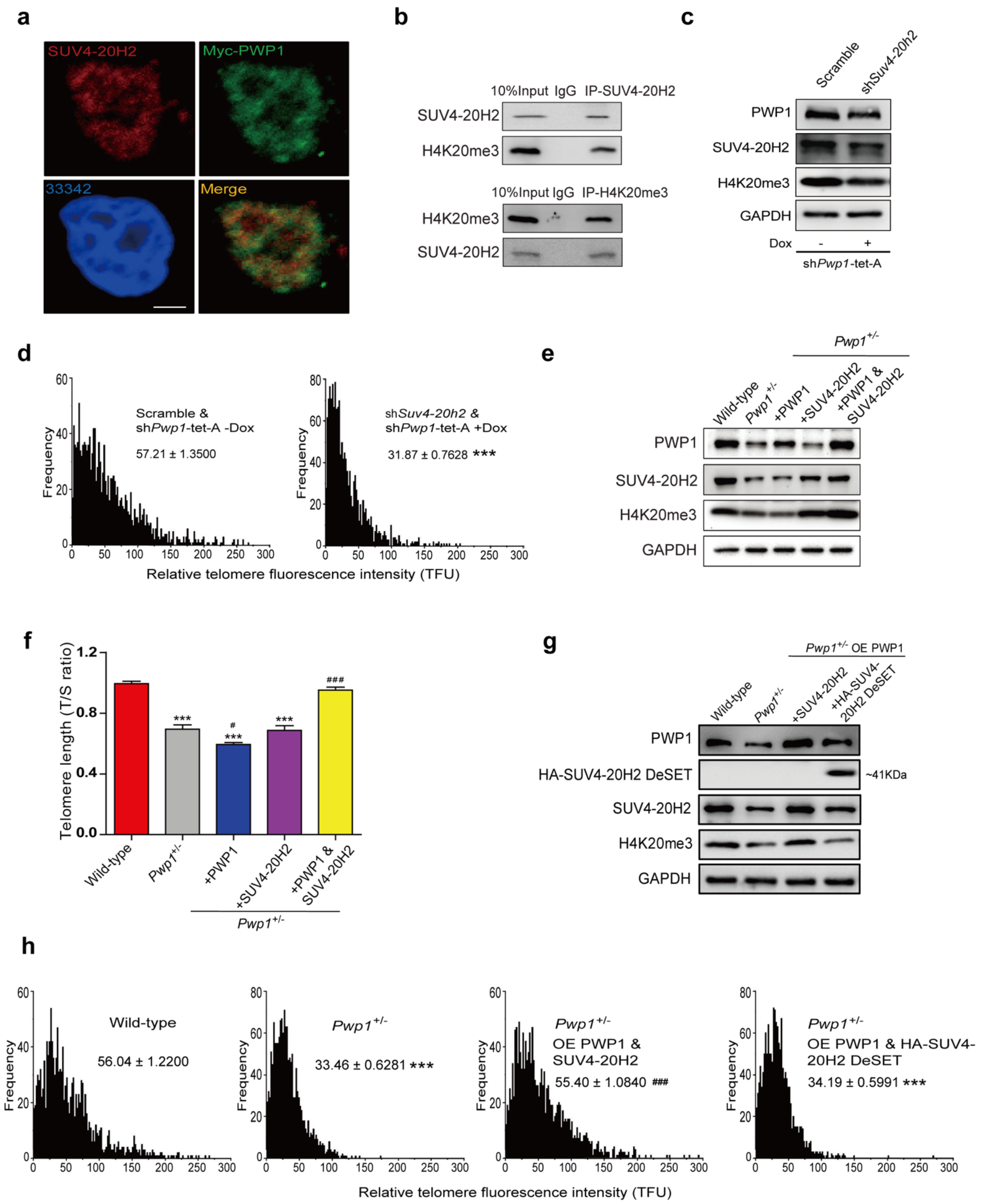

109

Supplementary Fig. S6: Rescue of telomere length in *Pwp1*-depleted ESCs. Related to Fig. 4.
a Co-localization of PWP1 and SUV4-20H2 in ESCs. Cells were stained for SUV4-20H2 (red), Myc-PWP1 (Myc; green), and nuclei (Hoechst 33342; blue). The scale bar represents 10 μm.
b Interaction between SUV4-20H2 and H4K20me3 proteins. Extracts from ESCs were immunoprecipitated using antibodies against SUV4-20H2 or H4K20me3 proteins, followed by Western blot analysis using both antibodies.
c Protein expression levels were determined by Western blot analysis in *Pwp1&Suv4-20h2* KD ESCs.
d Relative telomere lengths were determined by QFISH in *Pwp1&Suv4-20h2* KD ESCs. The data are shown as the mean TFU ± SEM of three independent experiments. *** $P < 0.001$.
e Overexpression of PWP1 and SUV4-20H2 proteins in *Pwp1*$^{+/-}$ ESCs. *Pwp1*$^{+/-}$ ESCs were infected with PWP1 or SUV4-20H2 lentivirus-overexpressing plasmids. Cell extracts were analyzed by Western blot using the antibodies indicated.
f Relative telomere length determined by qPCR analysis and shown as the T/S ratio in wild-type, *Pwp1*$^{+/-}$, *Pwp1*$^{+/-}$/PWP1-OE, *Pwp1*$^{+/-}$/SUV4-20H2-OE, and *Pwp1*$^{+/-}$/PWP1 & SUV4-20H2-OE ESCs. The data are presented as the mean ± SEM of three independent experiments. ***P < 0.001 compared with wild-type ESCs, $^{\#}$P < 0.05 and $^{\#\#\#}$P < 0.001 compared with Pwp1$^{+/-}$ ESCs.
g Western blot analysis in wild-type ESCs, *Pwp1*$^{+/-}$ ESCs, *Pwp1*$^{+/-}$ ESCs over-expressing PWP1 and SUV4-20H2 or HA-SUV4-20H2 DeSET (losing methyltransferase function).
h Relative telomere lengths determined by telomere Q-FISH analysis and shown as TFU in wild-type ESCs, *Pwp1*$^{+/-}$ ESCs, *Pwp1*$^{+/-}$ ESCs over-expressing PWP1 and SUV4-20H2 or HA-SUV4-20H2 DeSET. The data are presented as the mean ± SEM of three independent experiments. ***P < 0.001 compared with wild-type ESCs, $^{\#\#\#}$P < 0.001 compared with *Pwp1*$^{+/-}$ ESCs.

Supplementary Fig.S7

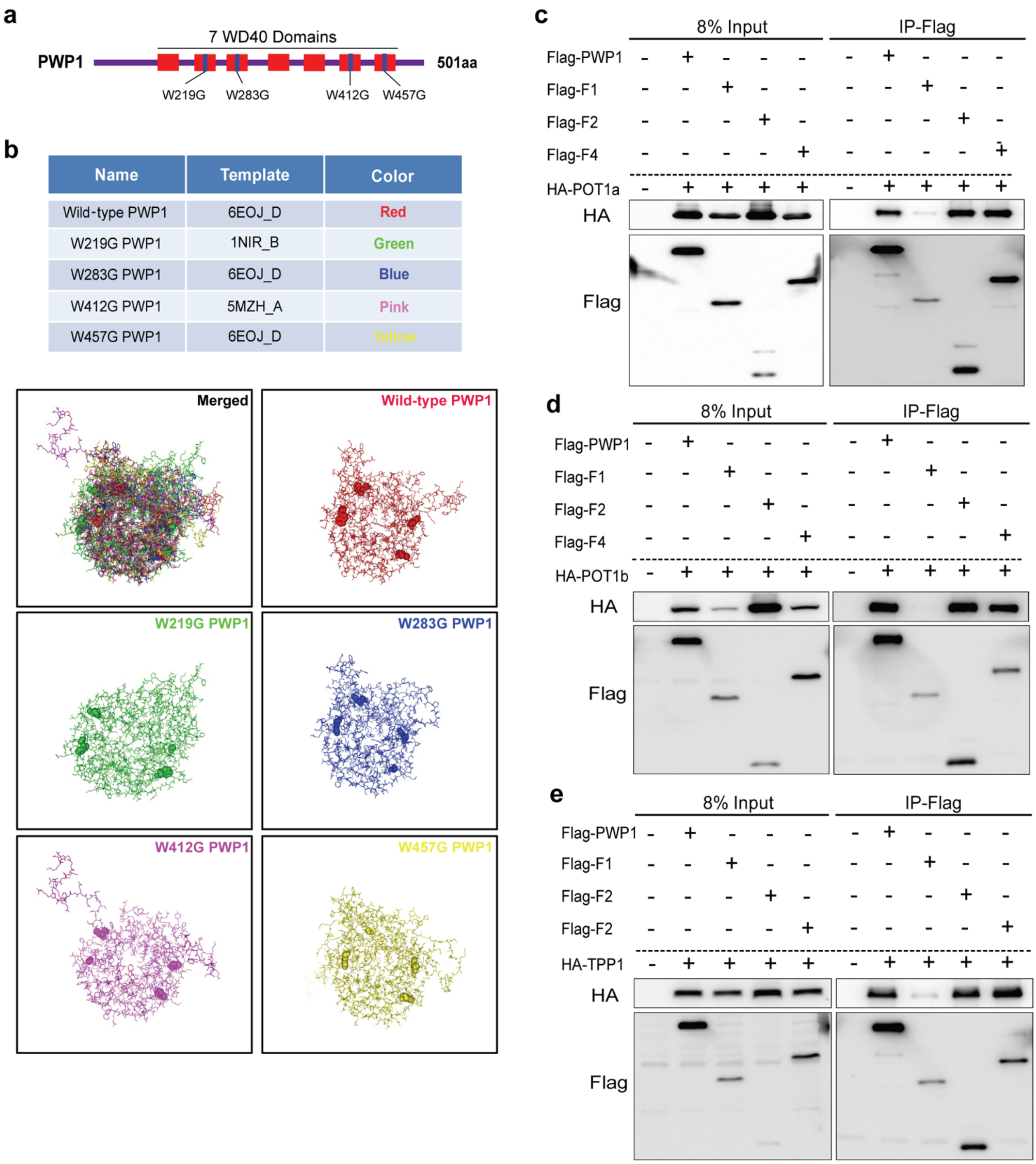

Supplementary Fig. S7: Binding between PWP1 fragments and shelterin complex. Related to Fig 5.

a Schematic representation of PWP1 mutants.

b Protein structural models of PWP1 mutants.

c-e Flag immunoprecipitation analysis in 293FT cells showing the binding between fragments of PWP1 (F1, F2, F4) and POT1a (**c**), POT1b (**d**), and TPP1 (**e**).

Supplementary Fig.S8

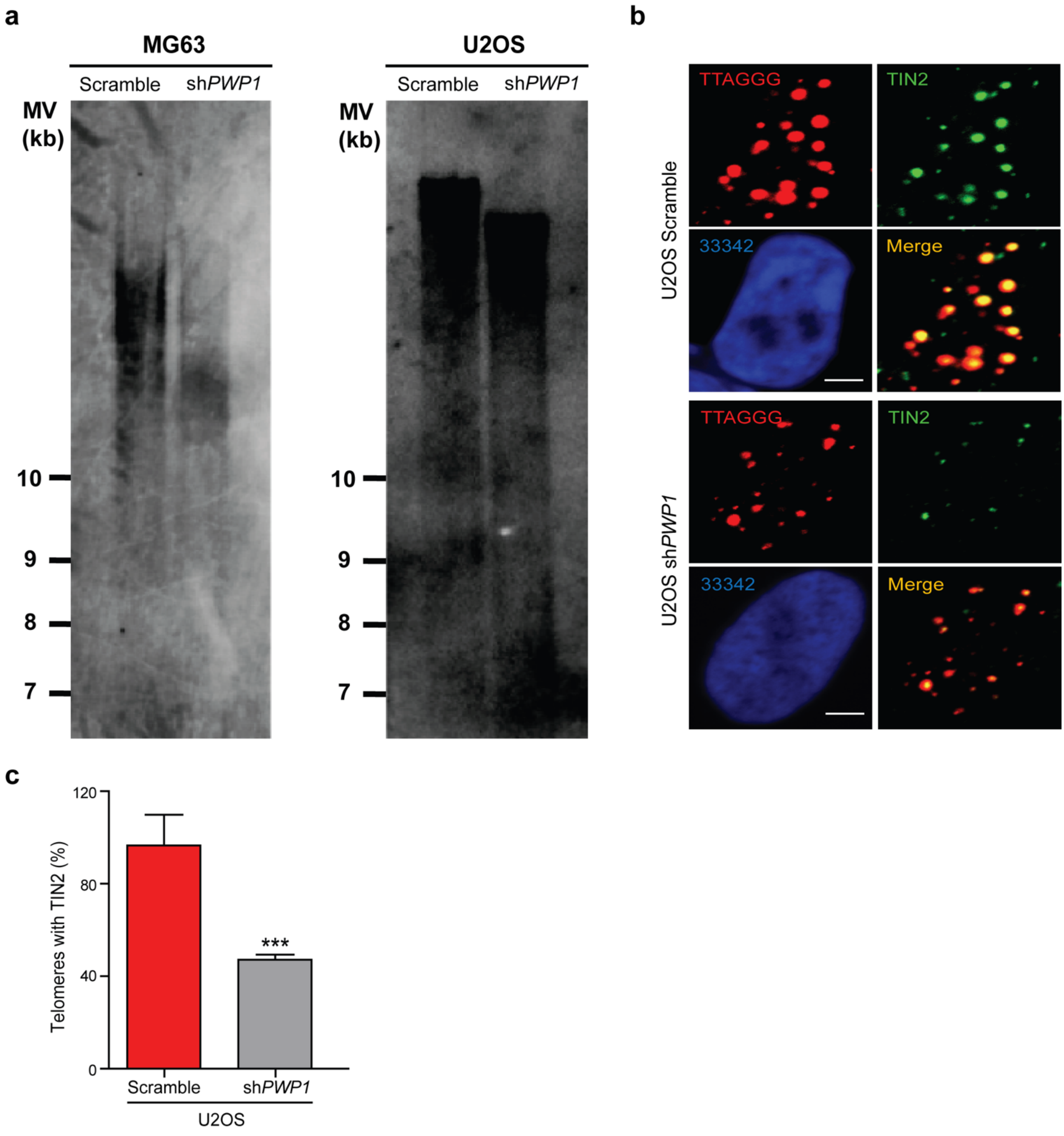

Supplementary Fig. S8: Telomere length and endogenous shelterin enrichment at the telomeres in *PWP1* KD human cells. Related to Fig. 6.

a Telomere restriction fragment (TRF) analysis upon *PWP1* knockdown in MG63 cells and U2OS cells.

b Representative images of endogenous shelterin protein (TIN2) localization in U2OS cells. Cells were stained for telomeres (TTAGGG; red), shelterin (TIN2; green), and nuclei (Hoechst 33342; blue). The scale bar represents 10 μm.

c Quantification of shelterin protein (TIN2) and telomere co-localizing foci. The data are presented as the mean ± SEM of three independent experiments. ***$P < 0.001$

Supplementary Tables

Supplementary Table S1. Sequences used in this study.

Notes	Num.	Name	Sequences
shRNA	1	shPwp1-tet-A F	CCGGGCTGAACAAGAGCAGTGCAATCTCGAGATTGCACTGCTCTTGTTCAGCTTTTTG
		shPwp1-tet-A R	AATTCAAAAAGCTGAACAAGAGCAGTGCAATCTCGAGATTGCACTGCTCTTGTTCAGC
	2	shZscan4-tet-A F	CCGGGACAGAAGCCTGGCATTCCCTCTCGAGAGGGAATGCCAGGCTTCTGTCTTTTTG
		shZscan4-tet-A R	AATTCAAAAAGACAGAAGCCTGGCATTCCCTCTCGAGAGGGAATGCCAGGCTTCTGTC
	3	shPwp1 F	CCGGGCTGAGTACGACTTAGATAAACTCGAGTTTATCTAAGTCGTACTCAGCTTTTTG
		shPwp1 R	AATTCAAAAAGCTGAGTACGACTTAGATAAACTCGAGTTTATCTAAGTCGTACTCAGC
	4	shSuv4-20h2 F	CCGGAGAGCTGATCCTTGTTCTATGCTCGAGCATAGAACAAGGATCAGCTCT TTTTTG
		shSuv4-20h2 R	AATTCAAAAA AGAGCTGATCCTTGTTCTATG CTCGAG CATAGAACAAGGATCAGCTCT
	5	shPot1 F	CCGGGCATTTCTCTACAACATTACTCGAGTAATGTTGTAGAGAAATGCTTTTTG
		shPot1 R	AATTCAAAAAGCATTTCTCTACAACATTACTCGAGTAATGTTGTAGAGAAATGC
	6	shTin2 F	CCGGCAGGAACTAGAACAAGACTATCTCGAGATAGTCTTGTTCTAGTTCCTGTTTTTG
		shTin2 R	AATTCAAAAACAGGAACTAGAACAAGACTATCTCGAG ATAGTCTTGTTCTAGTTCCTG
gRNA	7	Pwp1 gRNA F	GGCAAGCACCAATCCCTAACAC
		Pwp1 gRNA R	CCTCACATGGCTACAGTCCCG
	8	Pot1b gRNA F	CACCGTGTTCCACTTGTGACGTTGT
		Pot1b gRNA R	AAACACAACGTCACAAGTGGAACAG
	9	Fugw-Pwp1-Flag F	CGCGAATTCATGAACCGCAGCCGCCAAG

		Fugw-Pwp1-Flag R	CGCGGATCCTCAGGACTCCATTGGTGTCTGCTGTGGACTCCTGC
	10	Fugw-Pwp1-F1-Flag F	CGCGACCGGTATGGACTACAAGGACGACGATGACAAGAACCGCAGCCGCCAAGTGAC
		Fugw-Pwp1-F1-Flag R	CGCGGAATTCGTTTCCCACAGCAATGTAATTC
	11	Fugw-Pwp1-F2-Flag F	CGCGACCGGTATGGACTACAAGGACGACGATGACAAGACTGCACATACAGACAAGGTTCAG
		Fugw-Pwp1-F2-Flag R	CGCGGAATTCTCAGGACTCCATTGGTGTCTGCTGT
	12	Fugw-Pwp1-F3-Flag F	CGCGACCGGTATGGACTACAAGGACGACGATGACAAGAACCGCAGCCGCCAAGTGAC
		Fugw-Pwp1-F3-Flag R	CGCGGAATTCAAGCCTGGCTACTGACTTCCCCAC
	13	Fugw-Pwp1-F4-Flag F	CGCGACCGGTATGGACTACAAGGACGACGATGACAAGAACCGCAGCCGCCAAGTGAC
		Fugw-Pwp1-F4-Flag R	CGCGGAATTCTCAAAGCTTGCTTCCCAGTGTGA
	14	Fugw-Pwp1-Myc F	CGCGACCGGTATGGAGCAGAAACTCATCTCTGAAGAGGATCTGAACCGCAGCCGCCAAGTGAC
Overexpression		Fugw-Pwp1-Myc R	CGCGGAATTCTCAGGACTCCATTGGTGTCTGCTGT
	15	Fugw-Zscan4 F	CACAGACCGGTATGGCTTCACAGCAGGCACC
		Fugw-Zscan4 R	CACAGGAATTCTCAGTCAGATCTGTGGTAATTCCTC
	16	Fugw-Suv4-20h2-HA F	CACAGACCGGTATGTACCCATACGACGTCCCAGACTACGCTGGGCCTGATCGAGTGACAG
		Fugw-Suv4-20h2-HA R	CACAGGAATTCTCATGGCTCACCACTATTGATGTCC
	17	Fugw-Suv4-20h2 DeSET-HA F	GCGCCTTCCTGCCTGAGGAGGGCTTCTTCGGCGAGAAG
		Fugw-Suv4-20h2 DeSET-HA R	GAAGCCCTCCTCAGGCAGGAAGGCGCGG
	18	Fugw-Pot1a-HA F	CGCGACCGGTATGTACCCATACGACGTCCCAGACTACGCTTCTTTGGTTTCAACAGCTCCCTATAC
		Fugw-Pot1a-HA R	CGCGGAATTC CTAGACAACATTTTCTGCAACTG
	19	Fugw-Pot1b-HA F	CGCGACCGGTATGTACCCATACGACGTCCCAGACTACGCTTCTTCGGCCCCAGTAGCACCTTCTAC

		Fugw-Pot1b-HA R	CGCGGAATTCCTAGATGATGTCTTCTGCAATC
	20	Fugw-Trf1-HA F	CGCGACCGGTATGTACCCATACGACGTCCCAGACTACGCTGCGGAGACGGTCTCCTCAGCGGCCCG
		Fugw-Trf1-HA R	CGCGGAATTCTCAGCTAATCAGTTTCAGTCTC
	21	Fugw-Trf2-HA F	CGCGACCGGTATGTACCCATACGACGTCCCAGACTACGCTGCTGCGGGAGCCGGGACAGCGGGCC
		Fugw-Trf2-HA R	CGCGGAATTCTCATGGAAGTAAAACCAGGCCC
	22	Fugw-Tpp1-HA F	CGCGACCGGTATGTACCCATACGACGTCCCAGACTACGCTGGACTCCAAGCCCGCCTCCTAGGGC
		Fugw-Tpp1-HA R	CGCGGAATTCTCAAGGGTTGAGCAGGGTCTTC
	23	Fugw-Tin2-HA F	CGCGACCGGTATGTACCCATACGACGTCCCAGACTACGCTGCCCCACCTCCAGGGGTAGGTCCCG
		Fugw-Tin2-HA R	CGCGGAATTC TCACACTGTTCTCCTGCCTGCAG
	24	Fugw-Rap1-HA F	CACACGCGACCGGTATGTACCCATACGACGTCCCAGACTACGCTGCGGAGGCGATGGAT
		Fugw-Rap1-HA R	CACACAATTGTTATTTCTTTCGGAATTCAATCC
	25	Fugw-W219G-Flag F	CGAGGTAGGGGACCTGGATATAG
		Fugw-W219G-Flag R	CAGGTCCCCTACCTCGATGACAGG
	26	Fugw-W283G-Flag F	GTAGTTCTCGGGGATCTGTCTGTGGG
		Fugw-W283G-Flag R	ACAGATCCCCGAGAACTACAGTGCTG
	27	Fugw-W412G-Flag F	GTGAAGATCGGGGACATCTTAGGAG
		Fugw-W412G-Flag R	GATGTCCCCGATCTTCACAAATTTGTC
	28	Fugw-W457G-Flag F	CTTCGGGTTGGGGACATAAGCAC
		Fugw-W457G-Flag R	TATGTCCCCAACCCGAAGCCCTTC
	29	Fugw-Luciferase-Flag F	CGCGACCGGTATGGACTACAAGGACGACGATGACAAGAAGACGCCAAAAACATAAAGAAAGGCCC

		Fugw-Luciferase-Flag R	CGCGGAATTCATTACACGGCGATCTTTCC
	30	Fugw-Luciferase-HA F	CGCGACCGGTATGTACCCATACGACGTCCCAGACTACGCTAAGACGCCAAAAACATAAAGAAAGGCCC
		Fugw-Luciferase-HA R	CGCGGAATTCATTACACGGCGATCTTTCC
q-RT-PCR	31	Gapdh F	AGGTCGGTGTGAACGGATTTG
		Gapdh R	TGTAGACCATGTAGTTGAGGTCA
	32	Pwp1 F	AGAGACTCCAGACAAGGTAGAGC
		Pwp1 R	GTAATTCCCAGTAGATGCATCTG
	33	hPwp1 F	GGATGACAGGACGCTTGATGA
		hPwp1 R	CGTAGACCGTAAGACCCAAGA
	34	Terc F	GCTGTGGGTTCTGGTCTTTT
		Terc R	CTGCAGGTCTGGACTTTCCT
	35	Tert F	TTCTAGACTTGCAGGTGAACAGCC
		Tert R	TTCCTAACACGCTGGTCAAAGGGA
	36	hTert F	TGGATTTGCAGGTGAACAGCCTCCA
		hTert R	TGCAGCAGGAGGATCTTGTAGATG
	37	Pot1a F	GAAACTATGAAGCCCTCCCC
		Pot1a R	ATCCCTACAGTCCCTTCAAATG
	38	Pot1b F	CTAGTTATGGTCGTGGGATCAG
		Pot1b R	ATTGTCGGATTGGTAGGTGTC
	39	Tin2 F	GCGAAAATCCCGATCACATTG

		Tin2 R	AATGGGAGTAGCATGACTGTG
	40	Tpp1 F	CTTCAGCAATGTTTTCCCACG
		Tpp1 R	CAGTAGCCATCAGATAGTGCG
	41	Trf1 F	AGACGTGCTCCATCAGATTTC
		Trf1 R	TCTTCGGTTCCTTTCCATCG
q-RT-PCR for telomere	42	Tel F	CGGTTTGTTTGGGTTTGGGTTTGGGTTTGGGTTTGGGTT
		Tel R	GGCTTGCCTTACCCTTACCCTTACCCTTACCCTTACCCT
	43	36B4 F	ACTGGTCTAGGACCCGAGAAG
		36B4 R	TCAATGGTGCCTCTGGAGATT
	44	hTel F	CGGTTTGTTTGGGTTTGGGTTTGGGTTTGGGTTTGGGTT
		hTel R	GGCTTGCCTTACCCTTACCCTTACCCTTACCCTTACCCT
	45	hBg F	GCTTCTGACACAACTGTGTTCACTAGC
		hBg R	CACCAACTTCATCCACGTTCACC
ChIP-qPCR for subtelomere	46	ch1 subtel-F	GCAGAAGATTCCGGTTGTCC
		ch1 subtel-R	CAATGCCTTGCCTCAAACCT
	47	ch6 subtel-F	GCGCTCGGAAAAGATGCTAT
		ch6 subtel-R	CCCTATCTGACAGGCGTTCT
	48	ch7 subtel-F	TCTTCAGAGCTAGCCCACAA
		ch7 subtel-R	ACCAACGAAAACCAAGTCAGT
	49	ch8 subtel-F	AGGTTTGACTGTTAGGGCCA

		ch8 subtel-R	GTCAAAGCTTCAACCACCGT
	50	ch9 subtel-F	TCAGACCCAAGCTGAAGGAG
		ch9 subtel-R	AGACTTGCTCCATCTGCCTT
	51	ch10 subtel-F	CCTCATTTCAATCCTGGTGTCC
		ch10 subtel-R	CTGGTTAAGTCTCATGATGGGC
	52	ch11 subtel-F	TGCAACTGATTCTCTTGCGC
		ch11 subtel-R	CACACTCGGAGTCTTTTCGC
	53	ch13subtel-F	GCACACTTGGTGGGCTAAGAAGATG
		ch13 subtel-R	TTAAATCCTGACCAAAATGCCTGGC
	54	ch15 subtel-F	TGCAGAGACACACAGACACA
		ch15 subtel-R	TGGGACCAAGCTTAGCACTT

Supplementary Table S2. Antibodies used in this study.

No.	Name	Company	Cot.
1	GAPDH	Bioworld	AP0063
2	PWP1	Abcam	ab190795
3	PWP1	Santa Cruz Biotechnology	sc-166656
4	TERT	Santa Cruz Biotechnology	sc-7212
5	TERT	Abcam	ab104588
6	ZSCAN4	Thermo Fisher	PA5-20901
7	TIN2	Abcam	ab13791
8	POT1	Abcam	ab21382
9	TPP1	Abcam	ab195234
10	TRF1	Cell Signaling Technology	#3529

11	H4K20me3	Millipore	07-463
12	H3K9me3	Abclonal	07-442
13	SUV4-20H2	Abcam	ab91224
14	HA	Cell Signaling Technology	5017s
15	HA	Abcam	ab9110
16	Flag	Cell Signaling Technology	14793s
17	Myc	Abcam	ab9106
18	γ-H2A.X	Abcam	ab26350
19	γ-H2A.X	Bioworld	BS4760
20	Ms-HRP	Cell Signaling Technology	7076s
21	Rb-HRP	Cell Signaling Technology	7074s
22	Goat-HRP	Santa Cruz Biotechnology	sc-2768
23	IgG Rb	Millipore	12-370
24	IgG Ms	Millipore	12-371
25	Rat IgG 488	Invitrogen	A21208
26	Rat IgG 594	Invitrogen	A21207
27	Mus IgG 488	Invitrogen	A21202
28	Goat IgG 488	Invitrogen	A11055
